# A case study for application of DNA barcoding in identifying species of some imported frozen fish fillets in Egypt

**DOI:** 10.1038/s41598-026-58341-0

**Published:** 2026-06-26

**Authors:** Nermeen Y. Abass

**Affiliations:** Department of Agricultural Botany, Faculty of Agriculture Saba-Basha, Alexandria University, P.O. Box 21531, Alexandria City, Egypt

**Keywords:** Species identification, Catfish species, Fish fillets, COI gene, Taxonomic relationship, Ecology, Ecology, Genetics, Molecular biology, Zoology

## Abstract

Mislabeling of fish product is a persistent problem driven by economic incentives, complex supply chains, and weak regulations. It has significant consequences for the economy, public health, and marine conservation. The study aims to identify the fish species of some imported frozen fish fillets in Alexandria and Cairo, Egypt. Commercial samples were analyzed and sequencing of a short fragment of mitochondrial cytochrome oxidase subunit I (COI) gene. GenBank and the Barcode of Life Data System (BOLD) databases revealed definitive identity matches in the range of 99.85%–100% for all imported frozen fish fillets. Sequencing of COI gene revealed two different species in the fish fillets, basa fish *Pangasius bocourti* and Swai/Sutchi *Pangasianodon hypophthalmus*. The results showed that 66.7% of the basa fish had been replaced by Swai/Sutchi fish. The phylogenetic analysis showed the sequenced DNA similar to the NCBI database reference sequence. This study revealed that the local grocery store’s imported catfish fillets were mislabeled. However, it is highly recommended that larger samples should be used in further studies to cover a greater geographic area. Meanwhile, DNA barcoding is an excellent technique for detecting how fish products are labelled in Egypt, which is crucial for enforcing quality and consumer protection.

**Supplementary Information:**

The online version contains supplementary material available at 10.1038/s41598-026-58341-0.

## Introduction

Aquaculture plays a vital role in producing aquatic animals to meet the rising demand for protein^1,2^. The production of aquaculture worldwide hit a record 130.9 million tonnes in 2022 and expected to reach 205 million tonnes in 2032^3^ to meet rising demands. More than 90% of aquaculture production takes place in low- and middle-income nations, where it reduces food insecurity and provides a vital source of income^4^.

Catfish (order Siluriformes) are a diverse group of fish representing more than 3,000 species, 478 genera and 36 families^5^. By the end of 2030, the number of catfish species could reach 5,000^6^. Catfish are high tolerance to low dissolved oxygen, which may have been brought on by pollutants like heavy metals^7^. Because of their high market value^8^, rapid growth, and resistance to a wide range of environmental conditions^9–15^, catfish have been cultivated in nearly 90 countries worldwide^6^, such as (i) North African catfish *Clarias gariepinus* which is widely farmed in South Africa, Kenya, Mali, Nigeria, and Brazil, (ii) channel catfish *Ictalurus punctatus* primarily cultured in USA, Mexico, Cuba, Russia, and China, (iii) striped catfish *Pangasianodon hypophthalmus* which is primarily raised in Vietnam and Thailand.

*Pangasius* fish, mainly Swai/Sutchi *Pangasianodon hypophthalmus* and basa *Pangasius bocourti* belonging to the family Pangasiidae in the order Siluriformes of catfish^16^. They are presently mostly raised in intensive farming ponds, although Vietnamese and Cambodian farmers throughout the Mekong basin have been cage-raising them for decades^17^. Although basa grows much more slowly than Swai/Sutchi and takes twice as long to become commercially available, it has a better texture, taste and flavour. Because it cannot withstand contaminated water settings as well as Swai/Sutchi, basa is also less able to adapt to intensive farming practices^18^. *Pangasius* fish have a great deal of promise for the production of convenient fish products, including fillets, which are easy to fillet because they don’t have intramuscular pin bones^19^. It is usually prepared into frozen fillets for both domestic use and export to the United States, Middle East, and Europe.

Vietnam is currently the world’s third-largest producer of fisheries products^20^. Vietnamese catfish products have exported to 140 markets worldwide^21^. Vietnam’s exports of *pangasius* to various markets reached 191 million USD in 2024^22^ and reached 1.023 billion USD^23^. Egypt, United Arab Emirates, and Saudi Arabia are the top three markets for Vietnamese *pangasius* enterprises. By the end of 2021, Vietnam’s *pangasius* exports to the Middle East market had a total value of 56.32 million USD. *Pangasius* exports to Egypt reached 18.7 million USD, which was more than 32% of the total export value to the whole region^24^.

Commercial fish product mislabeling is a widespread issue that occurs all around the world. The majority of seafood consumers are not aware that this problem directly affects them and may potentially have detrimental effects on their health. When a product’s label doesn’t match its contents, it’s called mislabeling^25^. While intentional mislabeling is a more prevalent practice to boost profits and/or circumvent fishing regulations.

Import restrictions and labelling laws have had an impact on the species and origins of imported catfish, but they haven’t substantially reduced the volume of imports. Numerous anecdotal and proven cases exist when catfish species are mistakenly classified as either another catfish species or a higher value species. To improve the examination of imported fish products such as fish fillets, including verifying accurate species labelling, the government should create new laws^26^. Accurate identification of fish species in the markets is a growing concern due to the high incidence of species substitution at international level^27^.

DNA barcoding using COI gene sequences offer a reliable method for identifying known species and finding new ones by comparing sequence variation^28,29^. The COI gene’s extensive taxonomic coverage in validated databases, such as National Center for Biotechnology Information GenBank (NCBI GenBank) and Barcode of Life Data System (BOLD), makes it a common marker in animal metabarcoding^30^. DNA-based methodologies have revealed seafood mislabeling, checking for fraud across the worldwide supply chain and confirming the legitimacy of species^31–34^. In Egypt, commercial seafood mislabeling is a serious problem, especially in processed products like fish fillets where visual identification is challenging. Studies using DNA barcoding have shown high rates of species substitution, which are frequently motivated by financial incentives to market less valuable fish as premium kinds^33^.

The current study aimed to conduct an initial survey to identify fish species and detect mislabeling of some imported frozen fish fillets purchased from local grocery markets in Alexandria and Cairo, Egypt, by comparing the cytochrome oxidase subunit I (COI) gene sequences of frozen fish fillets against global genetic databases.

## Materials and methods

### Sample collection

Twelve samples of frozen fish fillets labeled as *Pangasius* were purchased, between May, 2024 and May, 2025, from local grocery markets in Alexandria and Cairo, Egypt. Details about each sample were recorded, including purchase location, name on the label, and country of origin labeling. Samples details are shown in Table 1.

**Table 1:** Samples of frozen fish fillets obtained for analysis, information stated on the label and collection sites.

Samples	On label	Processed fish product		Origin of product			Sampling location
Fish fillet_1	Basa	Frozen Fillets		Vietnam			Alexandria, Egypt
Fish fillet_2	Basa	Frozen Fillets		Vietnam			Alexandria, Egypt
Fish fillet_3	Basa	Frozen Fillets		Vietnam			Alexandria, Egypt
Fish fillet_4	Basa	Frozen Fillets		Vietnam			Alexandria, Egypt
Fish fillet_5	Basa	Frozen Fillets		Vietnam			Alexandria, Egypt
Fish fillet_6	Basa	Frozen Fillets		Vietnam			Alexandria, Egypt
Fish fillet_7	Basa	Frozen Fillets		Vietnam			Cairo, Egypt
Fish fillet_8	Basa	Frozen Fillets		Vietnam			Cairo, Egypt
Fish fillet_9	Basa	Frozen Fillets		Vietnam			Cairo, Egypt
Fish fillet_10	Basa	Frozen Fillets		Vietnam			Cairo, Egypt
Fish fillet_11	Basa	Frozen Fillets		Vietnam			Cairo, Egypt
Fish fillet_12	Basa	Frozen Fillets		Vietnam			Cairo, Egypt

### DNA extraction

The genomic DNA extractions from the muscle tissues were performed using the G-spinTM total DNA kit (Cat NO. 17045, iNtRON Biotechnology, Inc., Korea) following the manufacturer’s protocol, and stored at − 20 °C prior to further analysis. The quantity and quality of extracted DNA was measured using a NanoDrop 2000/2000c spectrophotometer (Manufactured by ThermoScientific™, USA) and 1.2% agarose gel electrophoresis. The A260/280 ratios for all isolated genomic DNA were ≥ 1.8. All isolated DNA were diluted to 200 ng.

### PCR amplification and sequencing

The mitochondrial COI gene was amplified using primer of FishF1 (5’ TCA ACC AAC CAC AAA GAC ATT GGC AC 3’) and FishR1 (5’ TAG ACT TCT GGG TGG CCA AAG AAT CA 3’), described by Ward et al.^35^. The amplification reactions were performed as described in the protocols of Ashour et al.^29^. PCR products were visualized on a 1.5% agarose gels, containing ethidium bromide (10 mg/ml) and the most intense products were selected and purified using the Qiagen gel extraction kit for sequencing. All sequencing was done by the Macrogen sequencing lab, Korea using the Life Technologies POP7 technology. The Abass et al.^10,13^ techniques were followed in the purification and sequencing of the amplified PCR products. Sequences were submitted to the GenBank database with accession numbers PX795194- PX795205 (Table 2).

**Table 2:** List of imported frozen fish fillets in Egyptian markets and fish species, and their GenBank accession numbers used to build multiple sequence alignment using Clustal Omega website in Fig. S3, and Phylogenetic tree in Figs. 2.

Name of Fish Species	GenBank Accession Number	References
*Clarias batrachus*	JF292297	NCBI GenBank
*Clarias gariepinus*	JF292311	NCBI GenBank
*Clarias macrocephalus*	JF292324	NCBI GenBank
*Cranoglanis bouderius*	JF292338	NCBI GenBank
*Devario aequipinnatus*	KJ590087	NCBI GenBank
Fish fillet_1	PX795194	This Study
Fish fillet_2	PX795195	This Study
Fish fillet_3	PX795196	This Study
Fish fillet_4	PX795197	This Study
Fish fillet_5	PX795198	This Study
Fish fillet_6	PX795199	This Study
Fish fillet_7	PX795200	This Study
Fish fillet_8	PX795201	This Study
Fish fillet_9	PX795202	This Study
Fish fillet_10	PX795203	This Study
Fish fillet_11	PX795204	This Study
Fish fillet_12	PX795205	This Study
*Hemibagrus macropterus*	JF292339	NCBI GenBank
*Ictalurus furcatus*	JF292368	NCBI GenBank
*Ictalurus punctatus*	JF292353	NCBI GenBank
*Pangasianodon hypophthalmus*	JF292393	NCBI GenBank
*Pangasius bocourti*	JF292415	NCBI GenBank

### Molecular and bioinformatics data analysis

All sequences were aligned using the Clustal Omega website (https://www.ebi.ac.uk/jdispatcher/msa/clustalo). Sample identification based on the COI barcode sequences similarity approach was carried out using two databases; GenBank and the Barcode of Life Data System (BOLD), https://v3.boldsystems.org. The highest percent pairwise identity of the consensus sequence from each sample blasted (BLASTN) against NCBI were compared to the percent specimen similarity scores of the consensus sequence from each species using FASTA sequences within the BOLD, accessed on Jan. 10^th^, 2026. The BLASTN software available from NCBI (http://www.ncbi.nlm.nih.gov) was used to analyze DNA sequences. Sequence similarity > 99% and query coverage = 100% were used as thresholds to identify fish species. The multiple sequence alignments were processed using the Clustal Omega website to illustrate the homologous relationship of each sample. Accession numbers (GenBank) for voucher species which were used to construct multiple sequence alignments are listed in Table 2. 21 sequences of COI gene aligned with one out-group as a root, *Devario aequipinnatus* to determine evolutionary relationships and identification of fish species. The MEGA version 12.0^36^ was used to construct the phylogenetic tree. Phylogenetic analysis was established using the Neighbor-Joining (NJ) and maximum parsimony (MP) methods^37^. The robustness of the tree was assessed by performing bootstrapping analysis with 1000 replicates^38^. The distance of the COI nucleotide bases between and within the fish species was estimated using the Kimura-2-parameter distance model (K2P)^39^.

## Results

### Identification of fish species using DNA barcoding

**Fig. 1:**
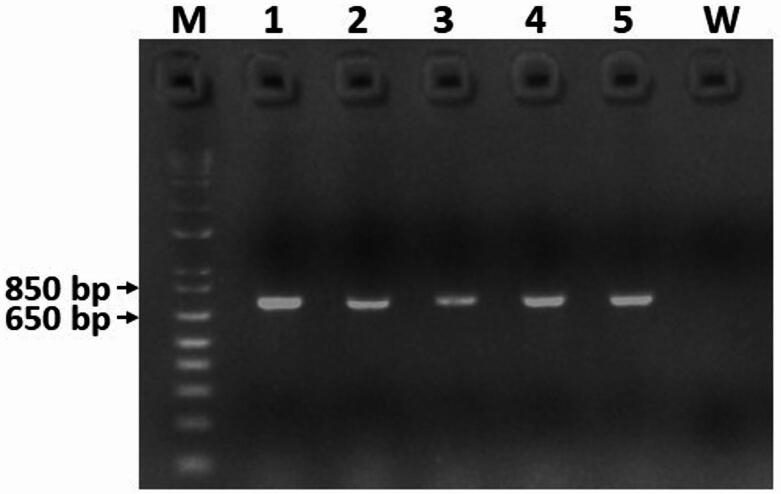
PCR analyses of the cytochrome oxidase subunit I (COI) gene of five representative imported fish fillets in Egyptian markets, total DNA. M = Marker, marker lanes contain 1 Kb plus DNA ladders (Invitrogen); 1 = Fish fillet_1; 2 = Fish fillet_2; 3 = Fish fillet_3; 4 = Fish fillet_4; 5 = Fish fillet_5; and W = Negative control (water).

For the information stated on the labels, all of them stated the common names, and the country of origin labeling. However, no trade market on the label (Table 1). The partial fragment (~ 650 bp) of mitochondrial cytochrome oxidase subunit I (COI) gene was successfully amplified using PCR from all the fish samples (Fig. 1). Table 3 shows the comprehensive barcoding identification results based on GenBank and the BOLD databases. The reference sequences of GenBank for the identification of all imported fish fillets were fish fillet_1 (JF292415), fish fillet_2 (JF292411), Fish fillet_3 (JF292419), fish fillet_4 (JF292425), fish fillet_5, and fish fillet_6 (PX312189), fish fillet_7, fish fillet_8, fish fillet_9, fish fillet_10, and fish fillet_12 (PP396129), and fish fillet_11 (MW829646) (Table 3). The BIN number of BOLD for the identification of fish fillets were fish fillet_1, fish fillet_2, fish fillet_3, and fish fillet_4 (BOLD: AAB7484), fish fillet_5, fish fillet_6, fish fillet_7, fish fillet_8, fish fillet_9, fish fillet_10, fish fillet_11, and fish fillet_12 (BOLD: AAE3237) (Table 3). The GenBank database revealed definitive identity matches in the range of 99.85%–100% for all imported frozen fish fillets and BOLD database revealed 100% identities at the species level. GenBank-based identification for all fish species yielded an alignment E-value of 0.0. At the time of analysis, GenBank results were in agreement with BOLD results in identification of all imported fish fillets. The GenBank and BOLD database hit using our fish fillet_1, fish fillet_2, fish fillet_3, and fish fillet_4 sequences was a single *Pangasius bocourti* sequence, while fish fillet_5, fish fillet_6, fish fillet_7, fish fillet_8, fish fillet_9, fish fillet_10, fish fillet_11, and fish fillet_12 was a single *Pangasianodon hypophthalmus* sequence. Fish fillet_1, fish fillet_5, fish fillet_6, fish fillet_7, fish fillet_8, fish fillet_9, fish fillet_10, fish fillet_11, and fish fillet_12 had 100% maximum identity in GenBank and BOLD database. Fish fillet_2, fish fillet_3, and fish fillet_4 had 99.85% maximum identity in GenBank and 100% maximum identity in BOLD database. Mislabeling was detected in the imported frozen fish fillets analyzed since all samples were genetically identified as *Pangasius bocourti* and *Pangasianodon hypophthalmus*. The results showed that 66.7% of the basa fish, *Pangasius bocourti* had been replaced by Swai/Sutchi fish, *Pangasianodon hypophthalmus*.

**Table 3:** Summary of frozen fish fillets samples identification based on each species consensus barcoded sequence using BLASTN search from GenBank and the Barcode of Life Data System (BOLD), and mislabeling (yes/no).

Samples	GenBank (BLASTN)					BOLD				Mislabelling
	Species identification	% Coverage	% Max identity	Accession number	Species identification		% Max Similarity	Sequence ID	BIN ID	
Fish fillet_1	*Pangasius bocourti*	100.00	100.00	JF292415	*Pangasius bocourti*		100.00	ANGBF8204-12	BOLD: AAB7484	NO
Fish fillet_2	*Pangasius bocourti*	100.00	99.85	JF292411	*Pangasius bocourti*		100.00	ANGBF8198-12	BOLD: AAB7484	NO
Fish fillet_3	*Pangasius bocourti*	100.00	99.85	JF292419	*Pangasius bocourti*		100.00	ANGBF8198-12	BOLD: AAB7484	NO
Fish fillet_4	*Pangasius bocourti*	100.00	99.85	JF292425	*Pangasius bocourti*		100.00	ANGBF8268-12	BOLD: AAB7484	NO
Fish fillet_5	*Pangasianodon hypophthalmus*	100.00	100.00	PX312189	*Pangasianodon hypophthalmus*		100.00	ANGBF43769-19	BOLD: AAE3237	Yes
Fish fillet_6	*Pangasianodon hypophthalmus*	100.00	100.00	PX312189	*Pangasianodon hypophthalmus*		100.00	ANGBF43769-19	BOLD: AAE3237	Yes
Fish fillet_7	*Pangasianodon hypophthalmus*	100.00	100.00	PP396129	*Pangasianodon hypophthalmus*		100.00	ANGBF43769-19	BOLD: AAE3237	Yes
Fish fillet_8	*Pangasianodon hypophthalmus*	100.00	100.00	PP396129	*Pangasianodon hypophthalmus*		100.00	ANGBF8209-12	BOLD: AAE3237	Yes
Fish fillet_9	*Pangasianodon hypophthalmus*	100.00	100.00	PP396129	*Pangasianodon hypophthalmus*		100.00	ANGBF43769-19	BOLD: AAE3237	Yes
Fish fillet_10	*Pangasianodon hypophthalmus*	100.00	100.00	PP396129	*Pangasianodon hypophthalmus*		100.00	ANGBF8209-12	BOLD: AAE3237	Yes
Fish fillet_11	*Pangasianodon hypophthalmus*	100.00	100.00	MW829646	*Pangasianodon hypophthalmus*		100.00	ANGBF43751-19	BOLD: AAE3237	Yes
Fish fillet_12	*Pangasianodon hypophthalmus*	100.00	100.00	PP396129	*Pangasianodon hypophthalmus*		100.00	ANGBF43769-19	BOLD: AAE3237	Yes

### Phylogenetic analysis

The COI gene sequences of the imported frozen fish fillets in the Egyptian markets were aligned using the Clustal Omega website (Supplementary, Fig. S1). Multiple sequence alignment of imported frozen fish fillets in Egyptian markets against reference sequences from GenBank, generated by Clustal Omega website are shown in (Supplementary, Fig. S2). From the COI gene sequence alignments, there were genetic variations at a nucleotide level (A, T, C, or G) as determined at different positions of the representative sequences. The current study’s nucleotide sequence data from the COI gene of different fish samples generated an alignment of ~ 650 bp. The fish species nucleotide discrimination revealed varied AT (adenine + thymine) and GC (guanine + cytosine) contents. Among the twelve representative fish samples, the observed nucleotide base composition of all analyzed sequences was 56.84% AT (range: 357–386) and 43.16% GC (range: 275–294) (Supplementary, Table S1).

**Fig. 2:**
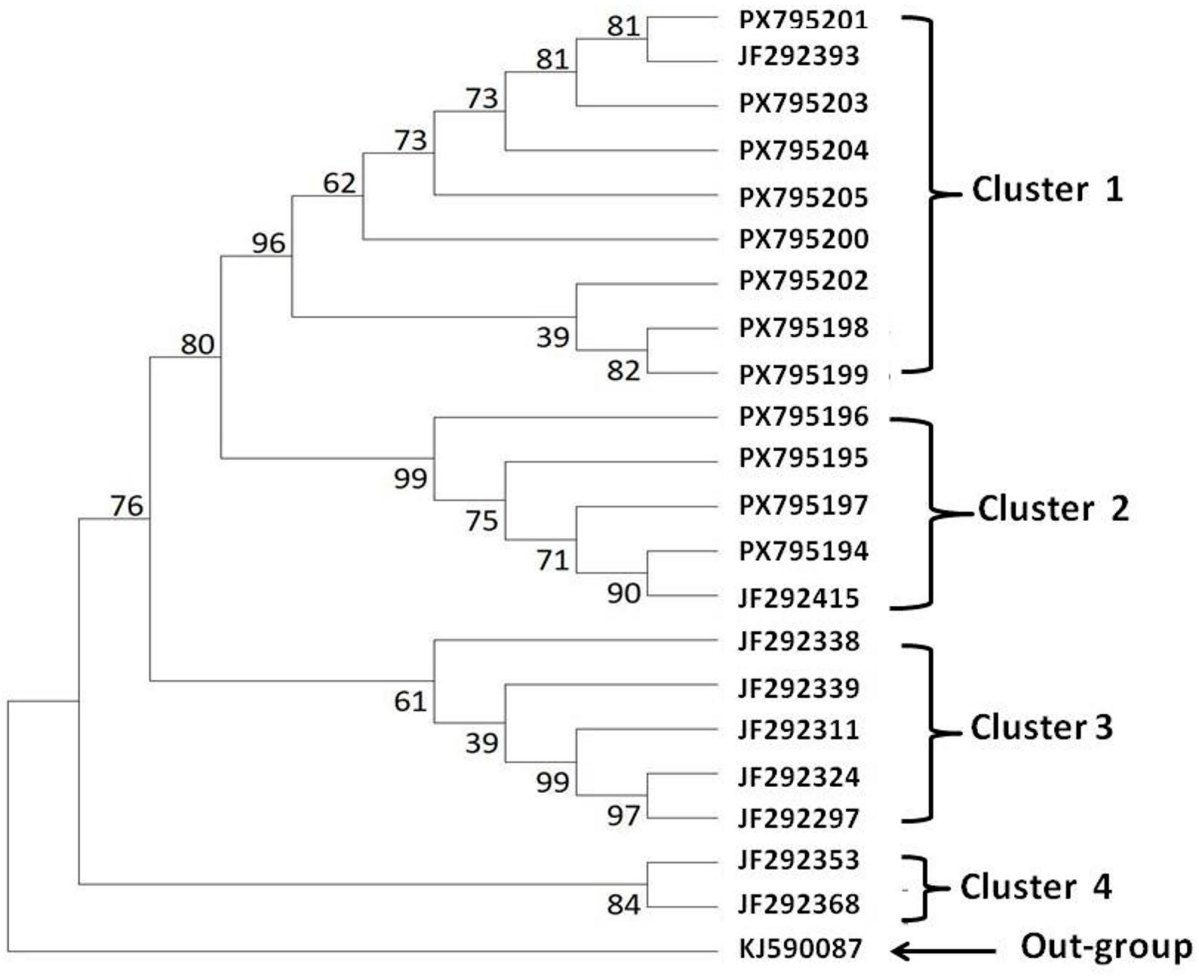
Phylogenetic tree of imported frozen fish fillets in Egyptian markets and catfish species sequences from GenBank prepared using MEGA version 12.0. Phylogenetic analyses of the fish species were performed based on Neighbor-Joining (NJ) and maximum parsimony (MP) methods. The % of replicate tree in which the associated taxa clustered together in the bootstrap test (1000 replicates) is shown next to the branches.

Nine catfish species namely walking catfish *Clarias batrachus*, North African catfish *Clarias gariepinus*, bighead catfish *Clarias macrocephalus*, helmet catfish *Cranoglanis bouderius*, long-barbel catfish *Hemibagrus macropteru*, blue catfish *Ictalurus furcatus*, channel catfish *Ictalurus punctatus*, swai or sutchi catfish *Pangasianodon hypophthalmu*, and basa catfish *Pangasius bocourti* were downloaded from NCBI GenBank (Table 2) to perform the phylogenetic analysis. The classification of the fish species used in this study is shown in Table 4. Classification, and scientific and common names of fish species were obtained from BOLD and Fishbase databases. The phylogenetic tree (Fig. 2) was constructed from the combined dataset of COI gene consisting of 21 sequences aligned with one out-group as a root, *Devario aequipinnatus* (Supplementary, Fig. S3).

**Table 4:** Classification of the fish species used in this study.

No	Specific name	Genus	Family	Order	Class	Common name
1	*Clarias batrachus*	*Clarias*	Clariidae	Siluriformes	Actinopterygii	Walking catfish
2	*Clarias gariepinus*	*Clarias*	Clariidae	Siluriformes	Actinopterygii	North African catfish
3	*Clarias macrocephalus*	*Clarias*	Clariidae	Siluriformes	Actinopterygii	Bighead catfish
4	*Cranoglanis bouderius*	*Cranoglanis*	Cranoglanididae	Siluriformes	Actinopterygii	Helmet catfish
5	*Hemibagrus macropterus*	*Hemibagrus*	Bagridae	Siluriformes	Actinopterygii	Long-barbel catfish
6	*Ictalurus furcatus*	*Ictalurus*	Ictaluridae	Siluriformes	Actinopterygii	Blue catfish
7	*Ictalurus punctatus*	*Ictalurus*	Ictaluridae	Siluriformes	Actinopterygii	Channel catfish
8	*Pangasianodon hypophthalmus*	*Pangasianodon*	Pangasiidae	Siluriformes	Actinopterygii	Swai or Sutchi catfish
9	*Pangasius bocourti*	*Pangasius*	Pangasiidae	Siluriformes	Actinopterygii	Basa catfish

From dendrogram it is clear that all genera of catfish were differentiated into 4 clusters. In cluster 1 the one species of the genus *Pangasianodon* was clustered with 96%, 81%, 73%, and 62% bootstrap values. In cluster 2 the one species of the genus *Pangasius* was clustered with 99%, 90%, 75%, and 71% bootstrap values. In cluster 3 the three species of the genus *Clarias*, *Cranoglanis*, and *Hemibagrus* were clustered with 99%, 97, 61%, and 39% bootstraps. In cluster 4 the one species of the genus *Ictalurus* was clustered with 84% bootstrap values. Cluster 1 and Cluster 2 consist of fishes of interest which shows that these fishes are from same lineage and must share a common ancestor. Genetic diversity among the twelve frozen fish fillets shows that fish fillet_1, fish fillet_2, fish fillet_3, and fish fillet_4 are closely related with *Pangasius bocourti* whereas fish fillet_5, fish fillet_6, fish fillet_7, fish fillet_8, fish fillet_9, fish fillet_10, fish fillet_11, and fish fillet_12 are related with *Pangasianodon hypophthalmus* (Fig. 2). The evolution of phylogenetic tree for all the fish species combined showed a clear distinction of the most fish families with most species of the same family were clustered together with observation of misplacement of species (Fig. 2).

The intraspecific and interspecies genetic distances were calculated with The Kimura-2-parameter model (K2P) to trace the evolutionary relationship between fish fillets samples, species, and families are summarized in Tables 5, 6, 7, 8 and 9. The minimum genetic distance between fish fillets samples (intraspecific genetic distances) was 0.00% and the maximum distance (interspecific genetic distances) was 0.12% (Table 5). The minimum genetic distance between fish species was 0.12 (between *Pangasianodon hypophthalmus* and *Pangasius bocourti*) and the maximum distance was 0.24 (between *Ictalurus furcatus* and *Clarias batrachus*) (Tables 6 and 7, and 9). The minimum value of genetic distance between fish families was 0.18 and the maximum value was 0.22 (Tables 8 and 9).

**Table 5:** Estimates of pairwise intraspecific genetic distances under Kimura-2-parameter distance model (K2P); in % differences for the cytochrome oxidase subunit I (COI) gene sequence between twelve representative imported frozen fish fillets samples in Egyptian markets.

No.	Species	Samples	1	2	3	4	5	6	7	8	9	10	11
1	*Pangasius bocourti*	Fish fillet_1											
2		Fish fillet_2	0.00										
3		Fish fillet_3	0.00	0.00									
4		Fish fillet_4	0.00	0.00	0.00								
5	*Pangasianodon hypophthalmus*	Fish fillet_5	0.12	0.12	0.11	0.11							
6		Fish fillet_6	0.12	0.12	0.11	0.11	0.00						
7		Fish fillet_7	0.12	0.12	0.11	0.11	0.00	0.00					
8		Fish fillet_8	0.12	0.12	0.12	0.11	0.00	0.00	0.00				
9		Fish fillet_9	0.12	0.12	0.11	0.11	0.00	0.00	0.00	0.00			
10		Fish fillet_10	0.12	0.12	0.12	0.11	0.00	0.00	0.00	0.00	0.00		
11		Fish fillet_11	0.12	0.12	0.12	0.11	0.00	0.00	0.00	0.00	0.00	0.00	
12		Fish fillet_12	0.12	0.12	0.11	0.11	0.00	0.00	0.00	0.00	0.00	0.00	0.00

**Table 6:** Estimates of pairwise genetic distances under Kimura-2-parameter distance model (K2P); in % differences for the cytochrome oxidase subunit I (COI) gene sequence between imported fish fillets in Egyptian markets and catfish species.

Species		1	2	3	4	5	6	7	8	9	10	11	12	13	14	15	16	17	18	19	20	21
1	Fish fillet_1																					
2	Fish fillet_2	0.00																				
3	Fish fillet_3	0.00	0.00																			
4	Fish fillet_4	0.00	0.00	0.00																		
5	Fish fillet_5	0.12	0.12	0.11	0.11																	
6	Fish fillet_6	0.12	0.12	0.11	0.11	0.00																
7	Fish fillet_7	0.12	0.12	0.11	0.11	0.00	0.00															
8	Fish fillet_8	0.12	0.12	0.11	0.11	0.00	0.00	0.00														
9	Fish fillet_9	0.12	0.12	0.11	0.11	0.00	0.00	0.00	0.00													
10	Fish fillet_10	0.12	0.12	0.11	0.11	0.00	0.00	0.00	0.00	0.00												
11	Fish fillet_11	0.12	0.12	0.11	0.11	0.00	0.00	0.00	0.00	0.00	0.00											
12	Fish fillet_12	0.12	0.12	0.11	0.11	0.00	0.00	0.00	0.00	0.00	0.00	0.00										
13	*Hemibagrus macropterus*	0.20	0.20	0.20	0.20	0.18	0.18	0.18	0.18	0.18	0.18	0.18	0.18									
14	*Devario aequipinnatus*	1.11	1.13	1.13	1.12	1.12	1.12	1.12	1.12	1.12	1.12	1.11	1.12	1.17								
15	*Ictalurus punctatus*	0.18	0.18	0.18	0.17	0.19	0.19	0.19	0.18	0.19	0.18	0.18	0.19	0.20	1.21							
16	*Ictalurus furcatus*	0.17	0.17	0.18	0.17	0.18	0.18	0.18	0.18	0.18	0.18	0.18	0.18	0.21	1.14	0.09						
17	*Pangasianodon hypophthalmus*	0.12	0.12	0.11	0.11	0.00	0.00	0.00	0.00	0.00	0.00	0.00	0.00	0.18	1.12	0.19	0.18					
18	*Pangasius bocourti*	0.00	0.00	0.00	0.00	0.12	0.12	0.12	0.12	0.12	0.12	0.12	0.12	0.20	1.11	0.18	0.17	0.12				
19	*Cranoglanis bouderius*	0.20	0.21	0.21	0.20	0.18	0.18	0.17	0.18	0.17	0.18	0.18	0.17	0.19	1.24	0.19	0.18	0.17	0.20			
20	*Clarias macrocephalus*	0.19	0.19	0.19	0.19	0.21	0.21	0.21	0.21	0.21	0.21	0.21	0.21	0.23	1.26	0.23	0.21	0.21	0.19	0.21		
21	*Clarias gariepinus*	0.20	0.20	0.20	0.20	0.21	0.21	0.21	0.21	0.21	0.21	0.21	0.21	0.21	1.33	0.20	0.22	0.21	0.20	0.21	0.15	
22	*Clarias batrachus*	0.22	0.22	0.21	0.23	0.25	0.25	0.24	0.24	0.24	0.24	0.25	0.24	0.22	1.29	0.23	0.24	0.24	0.22	0.23	0.14	0.16

**Table 7:** Estimates of pairwise genetic distances under Kimura-2-parameter distance model (K2P); in % differences for the cytochrome oxidase subunit I (COI) gene sequence between species of imported fish fillets in Egyptian markets and catfish species.

Species		1	2	3	4	5	6	7	8
1	*Pangasius bocourti*								
2	*Pangasianodon hypophthalmus*	0.12							
3	*Hemibagrus macropterus*	0.20	0.18						
4	*Ictalurus punctatus*	0.18	0.19	0.20					
5	*Ictalurus furcatus*	0.17	0.18	0.21	0.09				
6	*Cranoglanis bouderius*	0.20	0.18	0.19	0.19	0.18			
7	*Clarias macrocephalus*	0.19	0.21	0.23	0.23	0.21	0.21		
8	*Clarias gariepinus*	0.20	0.21	0.21	0.20	0.22	0.21	0.15	
9	*Clarias batrachus*	0.22	0.25	0.22	0.23	0.24	0.23	0.14	0.16

**Table 8:** Estimates of pairwise genetic distances under Kimura-2-parameter distance model (K2P); in % differences for the cytochrome oxidase subunit I (COI) gene sequence between families of imported fish fillets in Egyptian markets and catfish species.

Family		1	2	3	4
1	Pangasiidae				
2	Bagridae	0.19			
3	Ictaluridae	0.18	0.21		
4	Cranoglanis	0.19	0.19	0.18	
5	Clariidae	0.22	0.22	0.22	0.22

**Table 9:** Summary of genetic distances under Kimura-2-parameter distance model (K2P); in % differences for the cytochrome oxidase subunit I (COI) gene sequence within various taxonomic levels.

Comparisons within	Distance			Standard Deviation
	Mean	Minimum	Maximum	
Species	0.20	0.12	0.24	0.03
Families	0.20	0.18	0.22	0.02

## Discussion

Molecular species identification, especially DNA barcoding, offers a powerful solution for the expanding issue of fisheries and aquaculture mislabeling by providing accurate, rapid genetic “fingerprints” to verify the actual species of food products, helping to protect consumers, stop economic fraud, and aid in the conservation of threatened species^26^. DNA barcoding is a powerful, standardized technique for species identification that excels in speed and application throughout life stages. However, its limitations appear from its dependence on extensive reference databases and difficulties with specific taxonomic complexity, such as hybrids^40^. DNA barcoding is superior to other molecular markers in terms of universality, although it occasionally lacks the discriminating capacity of genome-wide approaches. In the current study, we successfully sequenced ~ 650 bp barcodes from the COI gene from all imported frozen fish fillets purchased from Alexandria and Cairo, Egypt. Since Alexandria and Cairo are Egypt’s biggest cities and main economic markets, they constitute an important cross-section of the country’s consumer market, making the choice of local grocery stores as the sampling regions extremely relevant. To cover a wider geographic area, it is strongly recommended that larger samples be used in future studies.

Comparison of COI gene sequences with sequences published in public databases (GenBank and BOLD) revealed 99.85–100% identities at the species level. In the present investigation, we were able to successfully sequence around 650 bp barcodes from the COI gene of imported frozen fish fillets that were bought from Egyptian marketplaces. Consequently, all of the imported fish fillets were correctly identified by the COI barcodes. According to the COI gene sequence, the fish species can be identified with the help of the DNA barcode^29,41–43^. According to our results, all sample of imported frozen fish fillets matched the reference sequences of COI gene in both databases (GenBank and the BOLD) and every species under investigation had a unique sequence that set them apart from one another.

Our results revealed that the average nucleotide base content was 56.84% AT and 43.16% GC. This result is consistent with previous studies that reported higher AT content than GC content with COI gene amplification in freshwater fish species^29,44–47^. The average GC% content for COI sequences gene for 30 freshwater fish sampled obtained from Lake Nasser and the River Nile was 46.44%^29^ and the average GC% content of 44 freshwater fish sampled obtained from Enugu and Anambra States was 45.78%^48^.

The COI gene sequences revealed the intraspecific genetic distances was 0.00% and the interspecific genetic distances was 0.12%. This finding is consistent with previous research showing that genetic distance within a species is smaller than that between species^29,49^. In Discogobio species, the average interspecific genetic distances were approximately 18.8 times greater than the average intraspecific genetic distances^28^. Our results showed that average genetic distance based on the K2P within species, and families were 0.20. Ashour et al.^29^ reported that the K2P values for 8 freshwater fish species collected from Lake Nasser and the River Nile were 0.227%, and 0.249% between species, and families, respectively. A more comprehensive understanding of the evolutionary history of catfish species can be obtained through taxonomic analysis and population genetics. A maximum genetic distance of 3% is adequate to distinguish the catfish species26. Species belonging to the same genera were, as expected, closely clustered into a single clade with a well-supported bootstrap proportion^50^. According to the Neighbour-joining tree, the dendrogram of the studied fish species and some imported frozen fish fillets samples revealed that the same cluster contained fishes of interest, indicating that these fishes must have a common ancestor and are from the same lineage. In a phylogenetic tree, low bootstrap values (< 70%) indicate uncertainty or weak signal. These are frequently caused by rapid evolution (short branches), contradicting data (incomplete lineage sorting, horizontal gene transfer), a lack of informative characters (short sequences, high similarity), or methodological problems (long-branch attraction), which indicate that the data does not strongly support that particular grouping over alternatives. It implies that, particularly if other branches have significant support, the node’s placement may be the result of artifacts or chance rather than compelling evolutionary evidence. The genetic diversity of the five fish fillet samples reveals that whereas fish fillet_5, fish fillet_6, fish fillet_7, fish fillet_8, fish fillet_9, fish fillet_10, fish fillet_11, and fish fillet_12 are related with Swai or Sutchi catfish *Pangasianodon hypophthalmus*, fish fillet_1, fish fillet_2, fish fillet_3, and fish fillet_4 are closely related to basa catfish *Pangasius bocourti*. The phylogenetic analysis showed the sequenced DNA similar to the NCBI database reference sequence. According to this study, the labeling on the imported catfish fillets that were sold in Egyptian local grocery stores was incorrect. This is consistent with the findings by Galal-Khallaf et al.^33^. The sequencing of COI gene of ninety commercial samples from Egyptian markets showed 33.3% species substitution in the fish fillets examined, 50% Nile perch *Lates niloticus* and 50% basa fish *Pangasius bocourti* being replaced by imported Vietnamese fish *Pangasianodon hypophthalmu*^33^. Similar to other global markets where seafood mislabeling has been exposed using DNA-based technology^32,51,52^. All imported fish must have an accurate product description and the country of origin written on the label in Arabic. According to Egyptian standards, such as those established by the Egyptian Organization for Standardization (EOS), using a “grade” or “type” that does not correspond with the biological species is deemed non-compliant. Mislabeling food items, such selling Swai as Basa, is considered a criminal violation under Egyptian law that involves “commercial fraud” and “misleading consumers.” The Consumer Protection Agency (CPA) and the National Food Safety Authority (NFSA) are responsible for enforcing penalties.

In contrast, Wong et al.^26^ reported that the labeling on the catfish fillets that were bought from the local grocery and oriental markets in USA was accurate. Pangasiidae was observed as sister group to Ictaluridae at a relatively low bootstrap percentage of 55%, whereas Cranoglanididae represented by *Cranoglanis bouderius* and Bagridae represented by *Hemibagrus macropterus* were the most diverged families from the rest of the groups and the mean genetic distance between these two families is 0.194%. Our findings are in line with those of Wong et al.^26^ has found that Pangasiidae was observed as sister group to Ictaluridae at a comparativaly low bootstrap percentage of 56%. In comparison to the other groups, Bagridae represented by *H. macropterus* was the most divergent^53^ and Funk and Omland^54^ also found that the clustering of *C. batrachus* and *C. macrocephalus* in one lineage and *C. gariepinus* in another lineage caused them to become geographically separated during the early phases of their evolution; with the former two catfish species being native Asian catfish and the latter of African origin. Ictaluridae are closely related to Pangasiidae, according to mitochondrial data^55^.

In conclusion, DNA barcoding and phylogenetic analysis provide Egypt with a reliable and rapid technique to identify commercial fish products, helping to prevent seafood fraud, where high-value fish species are illegally replaced with cheaper, lower-quality fish species for economic gain and safeguarding public health. DNA barcoding can accurately distinguish poisonous and harmful fish species and remove them from the market. By analyzing a short fragment of mitochondrial COI gene, regulatory authorities can confirm that the species on the label matches the product inside the packaging, even after processing. This molecular technique represents a significant improvement over traditional visual checks, which are often useless for processed fish such as fillets, fish balls, and canned fish. Economic incentives, morphological similarity in processed forms, market demand, and regulatory gaps all contribute to fish species substitution in imported frozen fish fillets in Egypt, especially within catfish species. *Pangasianodon hypophthalmus*, also known as swai or Sutchi catfish, is sometimes employed in substitute of more expensive basa, *Pangasius bocourti*, due to its quick growth, higher meat yield, and much cheaper market price.

## Supplementary Information

Below is the link to the electronic supplementary material.

Supplementary Material 1

Supplementary Material 2

## Data Availability

The datasets generated and analysed during the current study were deposited into the GenBank Barcode database under accession numbers PX795194- PX795205 and are available at the following URL: https://www.ncbi.nlm.nih.gov/nuccore/PX795194.1/- https://www.ncbi.nlm.nih.gov/nuccore/PX795205.1/.

## References

[1] FAO. Record fisheries and aquaculture production makes critical contribution to global food security. (2022). https://www.fao.org/newsroom/detail/record-fisheries-aquaculture-production-contributes-food-security-290622/en

[2] FAO. *The State of World Fisheries and Aquaculture 2022*. http://www.fao.org/documents/card/en/c/cc0461en (2022). 10.4060/cc0461en

[3] FAO. *FAO Report: Global fisheries and aquaculture production reaches a new record high*. (2025). https://www.fao.org/newsroom/detail/fao-report-global-fisheries-and-aquaculture-production-reaches-a-new-record-high/en

[4] Hambrey, J. The 2030 agenda and the sustainable development goals: the challenge for aquaculture development and management. FAO fisheries and aquaculture circular (C1141) (2017).

[5] Conservation, *Ecology, and Management of Catfish: The Second International Symposium *(American Fisheries Society, 2011). 10.47886/9781934874257

[6] Segaran, C. Catfishes: A global review of the literature. *Heliyon* **9**, e20081 (2023). 37810135 10.1016/j.heliyon.2023.e20081 PMC10559827

[7] Turan, F., Eken, M., Ozyilmaz, G., Karan, S. & Uluca, H. Heavy metal bioaccumulation, oxidative stress and genotoxicity in African catfish *Clarias gariepinus* from Orontes river. *Ecotoxicology* **29**, 1522–1537 (2020). 32710163 10.1007/s10646-020-02253-w

[8] Khalili Tilami, S. & Sampels, S. Nutritional value of fish: Lipids, proteins, vitamins, and minerals. *Rev. Fish. Sci. Aquac.* **26**, 243–253 (2018).

[9] Abass, N. et al. Genotype-environment interactions for growth and survival of channel catfish (*Ictalurus punctatus*), blue catfish (*Ictalurus furcatus*), and channel catfish, *I. punctatus*, ♀ × blue catfish, *I. furcatus*, ♂ hybrid fry at varying levels of sodium chloride. *Aquaculture.* **471**, 28–36 (2017).

[10] Abass, N. Y. et al. Genotype–environment interactions for survival at low and sub-zero temperatures at varying salinity for channel catfish, hybrid catfish and transgenic channel catfish. *Aquaculture* **458**, 140–148 (2016).

[11] Abass, N. Y. et al. Genotype-environment interactions for survival and growth rate at varying levels of sodium chloride for growth hormone transgenic channel catfish (*Ictalurus punctatus*), channel catfish, and albino channel catfish. *Aquaculture* **521**, 735084 (2020).

[12] Lisachov, A. et al. Emerging importance of bighead catfish (*Clarias macrocephalus*) and north African catfish (*C. gariepinus*) as a bioresource and their genomic perspective. *Aquaculture* **573**, 739585 (2023).

[13] Abass, N. Y., Ye, Z., Alsaqufi, A. & Dunham, R. A. Comparison of growth performance among channel-blue hybrid catfish, ccGH transgenic channel catfish, and channel catfish in a tank culture system. *Sci. Rep.* **12**740. (2022). 35031641 10.1038/s41598-021-04719-1 PMC8760261

[14] Abass, N. Y. et al. Effects of family and promoter on growth performance of ccGH cDNA transgenic channel catfish, *Ictalurus punctatus*, grown in a trough culture system. *Aquaculture* **536**, 736468 (2021).

[15] Abass, N. Y. et al. Growth differences of growth hormone transgenic female and male channel catfish, *Ictalurus punctatus*, grown in earthen ponds to sexual maturation. *Mar. Biotechnol.* **23**, 870–880 (2021). 10.1007/s10126-021-10069-w 34595591

[16] Wang, D. & Hsieh, Y. H. P. The use of imported pangasius fish in local restaurants. *Food Control*. **65**, 136–142 (2016).

[17] Binh, T. V., D’Haese, M., Speelman, S. & D’Haese, L. The influence of changes in the market environment on economic production characteristics of *pangasius* farming in the Mekong Delta (Vietnam). *Mar. Resour. Econ.* **25**, 373–390 (2010).

[18] Abd El-Fatah, R. A. et al. Improvement of microbial quality, physicochemical properties, fatty acids profile, and shelf life of basa (*Pangasius bocourti*) fillets during chilling storage using pepsin, rosemary oil, and citric acid. *Foods* **12**, 4170 (2023). 38002227 10.3390/foods12224170 PMC10670765

[19] U.S. International Trade Commission. Certain Frozen Fish Fillets from Vietnam. Investigation 731-TA-1012 (Review). (2009). https://www.usitc.gov/publications/701_731/pub4083.pdf

[20] VASEP. Vietnam-the third largest seafood exporter in the world. (2023). https://seafood.vasep.com.vn/why-buy-seafood/export-potentials/vietnam-the-third-largest-seafood-exporter-in-the-world-26061.html

[21] Bich Vo, T. T. Identification and analysis of snps in population of Vietnamese catfish (*Pangasianodon hypophthalmus*), using next generation sequencing and SNP validation. *MOJ Curr. Res. Rev.* **1**, 12–19 (2018).

[22] Hayati. Vietnam’s value-added pangasius export growth worldwide. (2024). https://mekseaconnection.com/vietnams-value-added-pangasius-export-growth-worldwide/

[23] VASEP. First half of 2025: Vietnam’s *pangasius* exports rise but growth potential tightens. (2025). https://seafood.vasep.com.vn/key-seafood-sectors/pangasius/news/first-half-of-2025-vietnam-s-pangasius-exports-rise-but-growth-potential-tightens-34239.html

[24] Hayati Egypt and UAE increase *pangasius* imports from Vietnam. (2021). https://mekseaconnection.com/egypt-and-uae-increase-pangasius-imports-from-vietnam/

[25] Ryburn, S. J. et al. Public awareness of seafood mislabeling. *PeerJ* **28**, e13486 (2022). 10.7717/peerj.13486 PMC9248779 35782099

[26] Wong, L. L. et al. DNA barcoding of catfish: species authentication and phylogenetic assessment. *PLoS One*. **6**, e17812 (2011). 21423623 10.1371/journal.pone.0017812 PMC3057997

[27] Acutis, P. L. et al. Detection of fish species substitution frauds in Italy: A targeted National Monitoring Plan. *Food Control*. **101**, 151–155 (2019).

[28] Li, H., Cheng, H., Huang, R., Qiu, Z. & Zhang, R. DNA barcoding of the genus Discogobio (Teleostei, Cyprinidae) in China. *Fishes* **10**, 157 (2025).

[29] Ashour, E. M., Ahmed, R. A. & Abass, N. Y. DNA barcoding and phylogenetic analysis to characterize biodiversity of some freshwater fish species in Lake Nasser and River Nile. *Sci. Rep.* **15**, 35237 (2025). 41068271 10.1038/s41598-025-20830-z PMC12511596

[30] Liu, K. et al. Application of DNA barcoding in fish identification of supermarkets in Henan province, China: More and longer COI gene sequences were obtained by designing new primers. *Food Res. Int.* **136**, 109516 (2020). 32846590 10.1016/j.foodres.2020.109516

[31] Von der Heyden, S., Barendse, J., Seebregts, A. J. & Matthee, C. A. Misleading the masses: detection of mislabelled and substituted frozen fish products in South Africa. *ICES J. Mar. Sci.* **67**, 176–185 (2010).

[32] Cutarelli, A. et al. Italian market fish species identification and commercial frauds revealing by DNA sequencing. *Food Control*. **37**, 46–50 (2014).

[33] Galal-Khallaf, A., Ardura, A., Mohammed-Geba, K., Borrell, Y. J. & Garcia-Vazquez, E. DNA barcoding reveals a high level of mislabeling in Egyptian fish fillets. *Food Control* **46**, 441–445 (2014).

[34] Pardo, M. Á. & Jiménez, E. DNA barcoding revealing seafood mislabeling in food services from Spain. *J. Food Compos. Anal.* **91**, 103521 (2020).

[35] Ward, R. D., Zemlak, T. S., Innes, B. H., Last, P. R. & Hebert, P. D. DNA barcoding Australia’s fish species. *Philos. Trans. R Soc. B Biol. Sci.* **360**, 1847–1857 (2005). 10.1098/rstb.2005.1716 PMC1609232 16214743

[36] Kumar, S. et al. MEGA12: molecular evolutionary genetic analysis version 12 for adaptive and green computing. *Mol Biol. Evol.* **41**, 12 (2024). 10.1093/molbev/msae263 PMC11683415 39708372

[37] Nei, M. & Kumar, S. *Molecular Evolution and Phylogenetics* (Oxford University Press, 2000).

[38] Felsenstein, J. Confidence limits on phylogenies: An approach using the bootstrap. *Evolution (N Y)* **39**, 783–791 (1985). 10.1111/j.1558-5646.1985.tb00420.x 28561359

[39] Kimura, M. A simple method for estimating evolutionary rates of base substitutions through comparative studies of nucleotide sequences. *J. Mol. Evol.* **16**, 111–120 (1980). 7463489 10.1007/BF01731581

[40] Antil, S. et al. DNA barcoding, an effective tool for species identification: a review. *Mol. Biol. Rep.* **50**, 761–775 (2023). 36308581 10.1007/s11033-022-08015-7

[41] Bingpeng, X. et al. DNA barcoding for identification of fish species in the Taiwan Strait. *PLoS One*. **13**, e0198109 (2018). 29856794 10.1371/journal.pone.0198109 PMC5983523

[42] Chang, C. et al. DNA barcodes of the native ray-finned fishes in Taiwan. *Mol. Ecol. Resour.* **17**, 796–805 (2017). 27717215 10.1111/1755-0998.12601

[43] Santana, P. et al. DNA barcode reveals occurrence of threatened species and hidden diversity on Teleost fish trade in the Coastal Amazon. *Sci. Rep.* **13**, 19749 (2023). 37957204 10.1038/s41598-023-47063-2 PMC10643451

[44] Nei, M. & Kumar, S. *Molecular Evolution and Phylogenetics* (Oxford University Press, 2000).

[45] Ghouri, M. Z. et al. Identification of edible fish species of Pakistan through DNA barcoding. *Front Mar. Sci.* **7**, 554183 (2020).

[46] Sajjad, A., Jabeen, F., Ali, M. & Zafar, S. DNA barcoding and phylogenetics of *Wallago attu* using mitochondrial COI gene from the River Indus. *J. King Saud Univ. - Sci.* **35**, 102725 (2023).

[47] Modeel, S. et al. A comprehensive DNA barcoding of Indian freshwater fishes of the Indus River system, Beas. *Sci. Rep.* **14**, 2763 (2024). 38307873 10.1038/s41598-024-52519-0 PMC10837433

[48] Ude, G. N. et al. DNA barcoding for identification of fish species from freshwater in Enugu and Anambra States of Nigeria. *Conserv. Genet. Resour.* **12**, 643–658 (2020).

[49] Mayer, I. & Pšenička, M. Conservation of teleost fishes: Application of reproductive technologies. *Theriogenology Wild*. **4**, 100078 (2024).

[50] Steinke, D., Zemlak, T. S. & Hebert, P. D. N. Barcoding nemo: DNA-based identifications for the ornamental fish trade. *PLoS One*. **4**, e6300 (2009). 19621079 10.1371/journal.pone.0006300 PMC2708913

[51] Senathipathi, D. N. et al. DNA barcoding revealed mislabeling of imported seafood products in Thailand. *Fishes* **9**, 215 (2024).

[52] Di Pinto, A. et al. DNA barcoding for detecting market substitution in salted cod fillets and battered cod chunks. *Food Chem.* **141**, 1757–1762 (2013). 23870888 10.1016/j.foodchem.2013.05.093

[53] Jondeung, A., Sangthong, P. & Zardoya, R. The complete mitochondrial DNA sequence of the Mekong giant catfish (*Pangasianodon gigas*), and the phylogenetic relationships among Siluriformes. *Gene* **387**, 49–57 (2007). 17067766 10.1016/j.gene.2006.08.001

[54] Funk, D. J. & Omland, K. E. Species-level paraphyly and polyphyly: frequency, causes, and consequences, with insights from animal mitochondrial DNA. *Annu. Rev. Ecol. Evol. Syst.* **34**, 397–423 (2003).

[55] Schedel, F. D. B. et al. New phylogenetic insights into the African catfish families Mochokidae and Austroglanididae. *J. Fish. Biol.* **100**, 1171–1186 (2022). 35184288 10.1111/jfb.15014 PMC9310817

