# Supplementary material for: A case study for application of DNA barcoding in identifying species of some imported frozen fish fillets in Egypt
Source: Sci Rep. 2026 Jun 26;16:19612. doi: 10.1038/s41598-026-58341-0 (PMC13309559; doi:10.1038/s41598-026-58341-0)
Supplement: Supplementary file 1 — Supplementary Material 1 [file 41598_2026_58341_MOESM1_ESM.docx]

**A case study for application of DNA barcoding in identifying species of some imported frozen fish fillets in Egypt**

Nermeen Y. Abass^a*^

a Department of Agricultural Botany, Faculty of Agriculture Saba-Basha, Alexandria University, Alexandria City, P.O. Box 21531, Egypt

*Corresponding author: + 2 03 5831646; fax: + 2 035832008

E-mail address: [n.y.abass@alexu.edu.eg](mailto:n.y.abass@alexu.edu.eg)

**PX795198 -----------------GCCTGAGCAGGAATAGTTGGTACGGCCCTCAGCCTCCTAATTC 43**

**PX795199 -----------------GCCTGAGCAGGAATAGTTGGTACGGCCCTCAGCCTCCTAATTC 43**

**PX795204 ---------------GTGCCTGAGCAGGAATAGTTGGTACGGCCCTCAGCCTCCTAATTC 45**

**PX795205 --------------GGTGCCTGAGCAGGAATAGTTGGTACGGCCCTCAGCCTCCTAATTC 46**

**PX795200 -----------------GCCTGAGCAGGAATAGTTGGTACGGCCCTCAGCCTCCTAATTC 43**

**PX795201 ---ACCTAGTATTTGGTGCCTGAGCAGGAATAGTTGGTACGGCCCTCAGCCTCCTAATTC 57**

**PX795202 -----------------GCCTGAGCAGGAATAGTTGGTACGGCCCTCAGCCTCCTAATTC 43**

**PX795203 ---ACCTAGTATTTGGTGCCTGAGCAGGAATAGTTGGTACGGCCCTCAGCCTCCTAATTC 57**

**PX795197 -----------------GCCTGAGCCGGAATAGTTGGTACAGCCCTTAGCCTGCTAATTC 43**

**PX795196 TCTATTTAGTGTTTGGTGCCTGAGCCGGAATAGTTGGTACAGCCCTTAGCCTCCTAATTC 60**

**PX795194 ---------TGTTTGGTGCCTGAGCCGGAATAGTTGGTACAGCCCTTAGCCTGCTAATTC 51**

**PX795195 ------TAGTGTTTGGTGCCTGAGCCGGAATAGTTGGTACAGCCCTTAGCCTGCTAATTC 54**

********** ************** ***** ***** *********

**PX795198 GGGCAGAGCTAGCCCAACCCGGCGCCCTTCTAGGCGACGACCAAATTTATAATGTTATTG 103**

**PX795199 GGGCAGAGCTAGCCCAACCCGGCGCCCTTCTAGGCGACGACCAAATTTATAATGTTATTG 103**

**PX795204 GGGCAGAGCTAGCCCAACCCGGCGCCCTTCTAGGCGACGACCAAATTTATAATGTTATTG 105**

**PX795205 GGGCAGAGCTAGCCCAACCCGGCGCCCTTCTAGGCGACGACCAAATTTATAATGTTATTG 106**

**PX795200 GGGCAGAGCTAGCCCAACCCGGCGCCCTTCTAGGCGACGACCAAATTTATAATGTTATTG 103**

**PX795201 GGGCAGAGCTAGCCCAACCCGGCGCCCTTCTAGGCGACGACCAAATTTATAATGTTATTG 117**

**PX795202 GGGCAGAGCTAGCCCAACCCGGCGCCCTTCTAGGCGACGACCAAATTTATAATGTTATTG 103**

**PX795203 GGGCAGAGCTAGCCCAACCCGGCGCCCTTCTAGGCGACGACCAAATTTATAATGTTATTG 117**

**PX795197 GGGCAGAGCTAGCCCAGCCCGGCGCCCTTCTGGGCGACGACCAAATTTATAATGTCATTG 103**

**PX795196 GGGCAGAGCTAGCCCAGCCCGGCGCCCTTCTGGGCGACGACCAAATTTATAATGTCATTG 120**

**PX795194 GGGCAGAGCTAGCCCAGCCCGGCGCCCTTCTGGGCGACGACCAAATTTATAATGTCATTG 111**

**PX795195 CGGCAGAGCTAGCCCAGCCCGGCGCCCTTCTGGGCGACGACCAAATTTATAATGTCATTG 114**

***************** ************** *********************** ******

**PX795198 TCACTGCCCATGCCTTCGTAATAATTTTCTTTATAGTAATACCAATTATAATTGGAGGCT 163**

**PX795199 TCACTGCCCATGCCTTCGTAATAATTTTCTTTATAGTAATACCAATTATAATTGGAGGCT 163**

**PX795204 TCACTGCCCATGCCTTCGTAATAATTTTCTTTATAGTAATACCAATTATAATTGGAGGCT 165**

**PX795205 TCACTGCCCATGCCTTCGTAATAATTTTCTTTATAGTAATACCAATTATAATTGGAGGCT 166**

**PX795200 TCACTGCCCATGCCTTCGTAATAATTTTCTTTATAGTAATACCAATTATAATTGGAGGCT 163**

**PX795201 TCACTGCCCATGCCTTCGTAATAATTTTCTTTATAGTAATACCAATTATAATTGGAGGCT 177**

**PX795202 TCACTGCCCATGCCTTCGTAATAATTTTCTTTATAGTAATACCAATTATAATTGGAGGCT 163**

**PX795203 TCACTGCCCATGCCTTCGTAATAATTTTCTTTATAGTAATACCAATTATAATTGGAGGCT 177**

**PX795197 TCACTGCCCATGCCTTCGTAATAATTTTCTTTATAGTAATACCAATTATGATTGGAGGCT 163**

**PX795196 TCACTGCCCATGCCTTCGTAATAATTTTCTTTATAGTAATACCAATTATGATTGGAGGCT 180**

**PX795194 TCACTGCCCATGCCTTCGTAATAATTTTCTTTATAGTAATACCAATTATGATTGGAGGCT 171**

**PX795195 TCACTGCCCATGCCTTCGTAATAATTTTCTTTATAGTAATACCAATTATGATTGGAGGCT 174**

*************************************************** ************

**PX795198 TTGGAAACTGACTTGTCCCCTTAATAATTGGAGCGCCTGATATGGCATTCCCTCGAATAA 223**

**PX795199 TTGGAAACTGACTTGTCCCCTTAATAATTGGAGCGCCTGATATGGCATTCCCTCGAATAA 223**

**PX795204 TTGGAAACTGACTTGTCCCCTTAATAATTGGAGCGCCTGATATGGCATTCCCTCGAATAA 225**

**PX795205 TTGGAAACTGACTTGTCCCCTTAATAATTGGAGCGCCTGATATGGCATTCCCTCGAATAA 226**

**PX795200 TTGGAAACTGACTTGTCCCCTTAATAATTGGAGCGCCTGATATGGCATTCCCTCGAATAA 223**

**PX795201 TTGGAAACTGACTTGTCCCCTTAATAATTGGAGCGCCTGATATGGCATTCCCTCGAATAA 237**

**PX795202 TTGGAAACTGACTTGTCCCCTTAATAATTGGAGCGCCTGATATGGCATTCCCTCGAATAA 223**

**PX795203 TTGGAAACTGACTTGTCCCCTTAATAATTGGAGCGCCTGATATGGCATTCCCTCGAATAA 237**

**PX795197 TCGGAAACTGACTTGTCCCCTTAATAATTGGAGCACCAGATATGGCATTTCCACGAATAA 223**

**PX795196 TCGGAAACTGACTTGTCCCCTTAATAATTGGAGCACCAGATATGGCATTTCCACGAATAA 240**

**PX795194 TCGGAAACTGACTTGTCCCCTTAATAATTGGGGCACCAGATATGGCATTTCCACGAATAA 231**

**PX795195 TCGGAAACTGACTTGTCCCCTTAATAATTGGAGCACCAGATATGGCATTTCCACGAATAA 234**

*** ***************************** ** ** *********** ** *********

**PX795198 ATAATATGAGTTTTTGATTACTTCCGCCTTCCTTCCTACTATTGCTTGCCTCCTCTGGAG 283**

**PX795199 ATAATATGAGTTTTTGATTACTTCCGCCTTCCTTCCTACTATTGCTTGCCTCCTCTGGAG 283**

**PX795204 ATAATATGAGTTTTTGATTACTTCCGCCTTCCTTCCTACTATTGCTTGCCTCCTCTGGAG 285**

**PX795205 ATAATATGAGTTTTTGATTACTTCCGCCTTCCTTCCTACTATTGCTTGCCTCCTCTGGAG 286**

**PX795200 ATAATATGAGTTTTTGATTACTTCCGCCTTCCTTCCTACTATTGCTTGCCTCCTCTGGAG 283**

**PX795201 ATAATATGAGTTTTTGATTACTTCCGCCTTCCTTCCTACTATTGCTTGCCTCCTCTGGAG 297**

**PX795202 ATAATATGAGTTTTTGATTACTTCCGCCTTCCTTCCTACTATTGCTTGCCTCCTCTGGAG 283**

**PX795203 ATAATATGAGTTTTTGATTACTTCCGCCTTCCTTCCTACTATTGCTTGCCTCCTCTGGAG 297**

**PX795197 ATAACATGAGTTTTTGATTACTCCCACCTTCCTTCCTACTACTGCTCGCCTCATCTGGAG 283**

**PX795196 ATAACATGAGTTTTTGATTACTCCCACCTTCCTTCCTACTACTGCTCGCCTCATCTGGAG 300**

**PX795194 ATAACATGAGTTTTTGATTACTCCCACCTTCCTTCCTACTACTGCTCGCCTCATCTGGAG 291**

**PX795195 ATAACATGAGTTTTTGATTACTCCCACCTTCCTTCCTACTACTGCTCGCCTCATCTGGAG 294**

****** ***************** ** *************** **** ***** *********

**PX795198 TAGAAGCAGGGGCAGGAACAGGATGAACTGTATATCCACCCCTTGCTGGAAACCTCGCAC 343**

**PX795199 TAGAAGCAGGGGCAGGAACAGGATGAACTGTATATCCACCCCTTGCTGGAAACCTCGCAC 343**

**PX795204 TAGAAGCAGGGGCAGGAACAGGATGAACTGTATATCCACCCCTTGCTGGAAACCTCGCAC 345**

**PX795205 TAGAAGCAGGGGCAGGAACAGGATGAACTGTATATCCACCCCTTGCTGGAAACCTCGCAC 346**

**PX795200 TAGAAGCAGGGGCAGGAACAGGATGAACTGTATATCCACCCCTTGCTGGAAACCTCGCAC 343**

**PX795201 TAGAAGCAGGGGCAGGAACAGGATGAACTGTATATCCACCCCTTGCTGGAAACCTCGCAC 357**

**PX795202 TAGAAGCAGGGGCAGGAACAGGATGAACTGTATATCCACCCCTTGCTGGAAACCTCGCAC 343**

**PX795203 TAGAAGCAGGGGCAGGAACAGGATGAACTGTATATCCACCCCTTGCTGGAAACCTCGCAC 357**

**PX795197 TTGAAGCGGGGGCAGGAACAGGATGAACTGTCTACCCACCTTTAGCCGGAAACCTAGCAC 343**

**PX795196 TTGAAGCGGGGGCAGGAACAGGATGAACTGTCTACCCACCTTTAGCCGGAAACCTAGCAC 360**

**PX795194 TTGAAGCGGGGGCAGGAACAGGATGAACTGTCTACCCACCTTTAGCCGGAAACCTAGCAC 351**

**PX795195 TTGAAGCGGGGGCAGGAACAGGATGAACTGTCTACCCACCTTTAGCCGGAAACCTAGCAC 354**

*** ***** *********************** ** ***** * ** ******** ******

**PX795198 ATGCCGGGGCTTCTGTAGATTTAACTATTTTCTCCCTTCATCTTGCAGGGGTATCATCCA 403**

**PX795199 ATGCCGGGGCTTCTGTAGATTTAACTATTTTCTCCCTTCATCTTGCAGGGGTATCATCCA 403**

**PX795204 ATGCCGGGGCTTCTGTAGATTTAACTATTTTCTCCCTTCATCTTGCAGGGGTATCATCCA 405**

**PX795205 ATGCCGGGGCTTCTGTAGATTTAACTATTTTCTCCCTTCATCTTGCAGGGGTATCATCCA 406**

**PX795200 ATGCCGGGGCTTCTGTAGATTTAACTATTTTCTCCCTTCATCTTGCAGGGGTATCATCCA 403**

**PX795201 ATGCCGGGGCTTCTGTAGATTTAACTATTTTCTCCCTTCATCTTGCAGGGGTATCATCCA 417**

**PX795202 ATGCCGGGGCTTCTGTAGATTTAACTATTTTCTCCCTTCATCTTGCAGGGGTATCATCCA 403**

**PX795203 ATGCCGGGGCTTCTGTAGATTTAACTATTTTCTCCCTTCATCTTGCAGGGGTATCATCCA 417**

**PX795197 ATGCCGGAGCTTCTGTAGATTTAACTATTTTCTCTCTTCACTTGGCAGGGGTATCATCTA 403**

**PX795196 ATGCCGGAGCTTCTGTAGATTTAACTATTTTCTCTCTTCACTTGGCAGGGGTATCATCTA 420**

**PX795194 ATGCCGGAGCTTCTGTAGATTTAACTATTTTCTCTCTTCACTTGGCAGGGGTATCATCTA 411**

**PX795195 ATGCCGGAGCTTCTGTAGATTTAACTATTTTCTCTCTTCACTTGGCAGGGGTATCATCTA 414**

********* ************************** ***** * ************** ***

**PX795198 TTCTAGGAGCCATTAATTTTATTACAACCATTATTAACATAAAACCACCAGCAATTTCAC 463**

**PX795199 TTCTAGGAGCCATTAATTTTATTACAACCATTATTAACATAAAACCACCAGCAATTTCAC 463**

**PX795204 TTCTAGGAGCCATTAATTTTATTACAACCATTATTAACATAAAACCACCAGCAATTTCAC 465**

**PX795205 TTCTAGGAGCCATTAATTTTATTACAACCATTATTAACATAAAACCACCAGCAATTTCAC 466**

**PX795200 TTCTAGGAGCCATTAATTTTATTACAACCATTATTAACATAAAACCACCAGCAATTTCAC 463**

**PX795201 TTCTAGGAGCCATTAATTTTATTACAACCATTATTAACATAAAACCACCAGCAATTTCAC 477**

**PX795202 TTCTAGGAGCCATTAATTTTATTACAACCATTATTAACATAAAACCACCAGCAATTTCAC 463**

**PX795203 TTCTAGGAGCCATTAATTTTATTACAACCATTATTAACATAAAACCACCAGCAATTTCAC 477**

**PX795197 TTCTAGGGGCCATTAACTTTATCACAACTATTATTAATATAAAACCCCCAGCAATTTCAC 463**

**PX795196 TTCTAGGGGCCATTAACTTTATCACAACTATTATTAATATAAAACCCCCAGCAATTTCAC 480**

**PX795194 TTCTAGGGGCCATTAACTTTATCACAACTATTATTAATATAAAACCCCCAGCAATTTCAC 471**

**PX795195 TTCTAGGGGCCATTAACTTTATCACAACTATTATTAATATAAAACCCCCAGCAATTTCAC 474**

********* ******** ***** ***** ******** ******** ***************

**PX795198 AATATCAAACACCTTTATTTGTATGGGCTGTCTTAATTACAGCTGTTCTTCTATTATTAT 523**

**PX795199 AATATCAAACACCTTTATTTGTATGGGCTGTCTTAATTACAGCTGTTCTTCTATTATTAT 523**

**PX795204 AATATCAAACACCTTTATTTGTATGGGCCGTCTTAATTACAGCTGTTCTTCTATTATTAT 525**

**PX795205 AATATCAAACACCTTTATTTGTATGGGCTGTCTTAATTACAGCTGTTCTTCTATTATTAT 526**

**PX795200 AATATCAAACACCTTTATTTGTATGGGCTGTCTTAATTACAGCTGTTCTTCTATTATTAT 523**

**PX795201 AATATCAAACACCTTTATTTGTATGGGCTGTCTTAATTACAGCTGTTCTTCTATTATTAT 537**

**PX795202 AATATCAAACACCTTTATTTGTATGGGCTGTCTTAATTACAGCTGTTCTTCTATTATTAT 523**

**PX795203 AATATCAAACACCTTTATTTGTATGGGCTGTCTTAATTACAGCTGTTCTTCTATTATTAT 537**

**PX795197 AGTACCAAACACCTCTATTTGTATGAGCTGTACTAATTACAGCTGTACTTCTACTGCTAT 523**

**PX795196 AGTACCAAACACCTCTATTTGTATGAGCTGTACTAATTACAGCTGTACTTCTACTGCTAT 540**

**PX795194 AGTACCAAACACCTCTATTTGTATGAGCTGTACTAATTACAGCTGTACTTCTACTGCTAT 531**

**PX795195 AGTACCAAACACCTCTATTTGTATGAGCTGTACTAATTACAGCTGTACTTCTACTGCTAT 534**

*** ** ********* ********** ** ** ************* ****** * *****

**PX795198 CTCTACCAGTACTGGCTGCCGGCATTACTATACTCCTAACAGATCGAAACCTAAATACTA 583**

**PX795199 CTCTACCAGTACTGGCTGCCGGCATTACTATACTCCTAACAGATCGAAACCTAAATACTA 583**

**PX795204 CTCTACCAGTACTGGCTGCCGGCATTACTATACTCCTAACAGATCGAAACCTAAATACTA 585**

**PX795205 CTCTACCAGTACTGGCTGCCGGCATTACTATACTCCTAACAGATCGAAACCTAAATACTA 586**

**PX795200 CTCTACCAGTACTGGCTGCCGGCATTACTATACTCCTAACAGATCGAAACCTAAATACTA 583**

**PX795201 CTCTACCAGTACTGGCTGCCGGCATTACTATACTCCTAACAGATCGAAACCTAAATACTA 597**

**PX795202 CTCTACCAGTACTGGCTGCCGGCATTACTATACTCCTAACAGATCGAAACCTAAATACTA 583**

**PX795203 CTCTACCAGTACTGGCTGCCGGCATTACTATACTCCTAACAGATCGAAACCTAAATACTA 597**

**PX795197 CTCTGCCCGTACTAGCCGCCGGCATTACAATACTTCTAACAGACCGAAATCTAAATACTA 583**

**PX795196 CTCTGCCCGTACTAGCCGCCGGCATTACAATACTTCTAACAGACCGAAATCTAAATACTA 600**

**PX795194 CTCTGCCCGTACTAGCCGCCGGCATTACAATACTTCTAACAGACCGAAATCTAAATACTA 591**

**PX795195 CTCTGCCCGTACTAGCCGCCGGCATTACAATACTTCTAACAGACCGAAATCTAAATACTA 594**

****** ** ***** ** *********** ***** ******** ***** ************

**PX795198 CATTCTTTGATCCTGCAGGCGGAGGAGATCCTATTTTATATCAACATCTTTTCTGATTCT 643**

**PX795199 CATTCTTTGATCCTGCAGGCGGAGGAGATCCTATTTTATATCAACATCTTTTCTGATTCT 643**

**PX795204 CATTCTTTGATCCTGCAGGCGGAGGAGATCCTATTTTATATCAACATCTTTTCTGATTCT 645**

**PX795205 CATTCTTTGATCCTGCAGGCGGAGGAGATCCTATTTTATATCAACATCTTTTCTGATTCT 646**

**PX795200 CATTCTTTGATCCTGCAGGCGGAGGAGATCCTATTTTATATCAACATCTTTTCTGATTCT 643**

**PX795201 CATTCTTTGATCCTGCAGGCGGAGGAGATCCTATTTTATATCAACATCTTTTCTGATTCT 657**

**PX795202 CATTCTTTGATCCTGCAGGCGGAGGAGATCCTATTTTATATCAACATCTTTTCTGATTCT 643**

**PX795203 CATTCTTTGATCCTGCAGGCGGAGGAGATCCTATTTTATATCAACATCTTTTCTGATTCT 657**

**PX795197 CATTCTTCGACCCCGCAGGGGGAGGAGACCCCATCCTATATCAACAGCTTTTCTGATTCT 643**

**PX795196 CATTCTTCGACCCCGCAGGGGGAGGAGACCCCATCCTATATCAACACCTTT--------- 651**

**PX795194 CATTCTTCGACCCCGCAGGGGGAGGAGACCCCATCCTATATCAACACCTTTTCTGATTCT 651**

**PX795195 CATTCTTCGACCCCGCAGGGGGAGGAGACCCCATCCTATATCAACACCTTTTCTGAT--- 651**

********* ** ** ***** ******** ** ** ********** ******

**PX795198 TCCGTCAC--- 651**

**PX795199 TCCGTCAC--- 651**

**PX795204 TCGGTCACCC- 655**

**PX795205 TCGGT------ 651**

**PX795200 TCGGTCAC--- 651**

**PX795201 TCGGTCACCCT 668**

**PX795202 TCGGTCAC--- 651**

**PX795203 TCGGTCACCCT 668**

**PX795197 TCGGTCAC--- 651**

**PX795196 ----------- 651**

**PX795194 ----------- 651**

**PX795195 ----------- 651**

**Figure S1.** Multiple sequence alignment of imported fish fillets in Egyptian markets, generated by Clustal Omega. Identities and similarities are indicated by star (*) and gaps are indicated by dashes (-).

**PX795198 -----------------GCCTGAGCAGGAATAGTTGGTACGGCCCTCAGCCTCCTAATTC 43**

**PX795199 -----------------GCCTGAGCAGGAATAGTTGGTACGGCCCTCAGCCTCCTAATTC 43**

**PX312189 -----------------GCCTGAGCAGGAATAGTTGGTACGGCCCTCAGCCTCCTAATTC 43**

**PX795204 ---------------GTGCCTGAGCAGGAATAGTTGGTACGGCCCTCAGCCTCCTAATTC 45**

**MW829646 ---------------GTGCCTGAGCAGGAATAGTTGGTACGGCCCTCAGCCTCCTAATTC 45**

**PX795205 --------------GGTGCCTGAGCAGGAATAGTTGGTACGGCCCTCAGCCTCCTAATTC 46**

**PX795200 -----------------GCCTGAGCAGGAATAGTTGGTACGGCCCTCAGCCTCCTAATTC 43**

**PX795201 ---ACCTAGTATTTGGTGCCTGAGCAGGAATAGTTGGTACGGCCCTCAGCCTCCTAATTC 57**

**PX795202 -----------------GCCTGAGCAGGAATAGTTGGTACGGCCCTCAGCCTCCTAATTC 43**

**PX795203 ---ACCTAGTATTTGGTGCCTGAGCAGGAATAGTTGGTACGGCCCTCAGCCTCCTAATTC 57**

**JF292393 -----------------GCCTGAGCAGGAATAGTTGGTACGGCCCTCAGCCTCCTAATTC 43**

**PP396129 ---ACCTAGTATTTGGTGCCTGAGCAGGAATAGTTGGTACGGCCCTCAGCCTCCTAATTC 57**

**PX795197 -----------------GCCTGAGCCGGAATAGTTGGTACAGCCCTTAGCCTGCTAATTC 43**

**JF292425 -----------------GCCTGAGCCGGAATAGTTGGTACAGCCCTTAGCCTGCTAATTC 43**

**PX795196 TCTATTTAGTGTTTGGTGCCTGAGCCGGAATAGTTGGTACAGCCCTTAGCCTCCTAATTC 60**

**JF292419 TCTATTTAGTGTTTGGTGCCTGAGCCGGAATAGTTGGTACAGCCCTTAGCCTGCTAATTC 60**

**PX795194 ---------TGTTTGGTGCCTGAGCCGGAATAGTTGGTACAGCCCTTAGCCTGCTAATTC 51**

**JF292415 ---------TGTTTGGTGCCTGAGCCGGAATAGTTGGTACAGCCCTTAGCCTGCTAATTC 51**

**PX795195 ------TAGTGTTTGGTGCCTGAGCCGGAATAGTTGGTACAGCCCTTAGCCTGCTAATTC 54**

**JF292411 ------TAGTGTTTGGTGCCTGAGCCGGAATAGTTGGTACAGCCCTTAGCCTGCTAATTC 54**

********** ************** ***** ***** *********

**PX795198 GGGCAGAGCTAGCCCAACCCGGCGCCCTTCTAGGCGACGACCAAATTTATAATGTTATTG 103**

**PX795199 GGGCAGAGCTAGCCCAACCCGGCGCCCTTCTAGGCGACGACCAAATTTATAATGTTATTG 103**

**PX312189 GGGCAGAGCTAGCCCAACCCGGCGCCCTTCTAGGCGACGACCAAATTTATAATGTTATTG 103**

**PX795204 GGGCAGAGCTAGCCCAACCCGGCGCCCTTCTAGGCGACGACCAAATTTATAATGTTATTG 105**

**MW829646 GGGCAGAGCTAGCCCAACCCGGCGCCCTTCTAGGCGACGACCAAATTTATAATGTTATTG 105**

**PX795205 GGGCAGAGCTAGCCCAACCCGGCGCCCTTCTAGGCGACGACCAAATTTATAATGTTATTG 106**

**PX795200 GGGCAGAGCTAGCCCAACCCGGCGCCCTTCTAGGCGACGACCAAATTTATAATGTTATTG 103**

**PX795201 GGGCAGAGCTAGCCCAACCCGGCGCCCTTCTAGGCGACGACCAAATTTATAATGTTATTG 117**

**PX795202 GGGCAGAGCTAGCCCAACCCGGCGCCCTTCTAGGCGACGACCAAATTTATAATGTTATTG 103**

**PX795203 GGGCAGAGCTAGCCCAACCCGGCGCCCTTCTAGGCGACGACCAAATTTATAATGTTATTG 117**

**JF292393 GGGCAGAGCTAGCCCAACCCGGCGCCCTTCTAGGCGACGACCAAATTTATAATGTTATTG 103**

**PP396129 GGGCAGAGCTAGCCCAACCCGGCGCCCTTCTAGGCGACGACCAAATTTATAATGTTATTG 117**

**PX795197 GGGCAGAGCTAGCCCAGCCCGGCGCCCTTCTGGGCGACGACCAAATTTATAATGTCATTG 103**

**JF292425 GGGCAGAGCTAGCCCAGCCCGGCGCCCTTCTGGGCGACGACCAAATTTATAATGTCATTG 103**

**PX795196 GGGCAGAGCTAGCCCAGCCCGGCGCCCTTCTGGGCGACGACCAAATTTATAATGTCATTG 120**

**JF292419 GGGCAGAGCTAGCCCAGCCCGGCGCCCTTCTGGGCGACGACCAAATTTATAATGTCATTG 120**

**PX795194 GGGCAGAGCTAGCCCAGCCCGGCGCCCTTCTGGGCGACGACCAAATTTATAATGTCATTG 111**

**JF292415 GGGCAGAGCTAGCCCAGCCCGGCGCCCTTCTGGGCGACGACCAAATTTATAATGTCATTG 111**

**PX795195 CGGCAGAGCTAGCCCAGCCCGGCGCCCTTCTGGGCGACGACCAAATTTATAATGTCATTG 114**

**JF292411 GGGCAGAGCTAGCCCAGCCCGGCGCCCTTCTGGGCGACGACCAAATTTATAATGTCATTG 114**

***************** ************** *********************** ******

**PX795198 TCACTGCCCATGCCTTCGTAATAATTTTCTTTATAGTAATACCAATTATAATTGGAGGCT 163**

**PX795199 TCACTGCCCATGCCTTCGTAATAATTTTCTTTATAGTAATACCAATTATAATTGGAGGCT 163**

**PX312189 TCACTGCCCATGCCTTCGTAATAATTTTCTTTATAGTAATACCAATTATAATTGGAGGCT 163**

**PX795204 TCACTGCCCATGCCTTCGTAATAATTTTCTTTATAGTAATACCAATTATAATTGGAGGCT 165**

**MW829646 TCACTGCCCATGCCTTCGTAATAATTTTCTTTATAGTAATACCAATTATAATTGGAGGCT 165**

**PX795205 TCACTGCCCATGCCTTCGTAATAATTTTCTTTATAGTAATACCAATTATAATTGGAGGCT 166**

**PX795200 TCACTGCCCATGCCTTCGTAATAATTTTCTTTATAGTAATACCAATTATAATTGGAGGCT 163**

**PX795201 TCACTGCCCATGCCTTCGTAATAATTTTCTTTATAGTAATACCAATTATAATTGGAGGCT 177**

**PX795202 TCACTGCCCATGCCTTCGTAATAATTTTCTTTATAGTAATACCAATTATAATTGGAGGCT 163**

**PX795203 TCACTGCCCATGCCTTCGTAATAATTTTCTTTATAGTAATACCAATTATAATTGGAGGCT 177**

**JF292393 TCACTGCCCATGCCTTCGTAATAATTTTCTTTATAGTAATACCAATTATAATTGGAGGCT 163**

**PP396129 TCACTGCCCATGCCTTCGTAATAATTTTCTTTATAGTAATACCAATTATAATTGGAGGCT 177**

**PX795197 TCACTGCCCATGCCTTCGTAATAATTTTCTTTATAGTAATACCAATTATGATTGGAGGCT 163**

**JF292425 TCACTGCCCATGCCTTCGTAATAATTTTCTTTATAGTAATACCAATTATGATTGGAGGCT 163**

**PX795196 TCACTGCCCATGCCTTCGTAATAATTTTCTTTATAGTAATACCAATTATGATTGGAGGCT 180**

**JF292419 TCACTGCCCATGCCTTCGTAATAATTTTCTTTATAGTAATACCAATTATGATTGGAGGCT 180**

**PX795194 TCACTGCCCATGCCTTCGTAATAATTTTCTTTATAGTAATACCAATTATGATTGGAGGCT 171**

**JF292415 TCACTGCCCATGCCTTCGTAATAATTTTCTTTATAGTAATACCAATTATGATTGGAGGCT 171**

**PX795195 TCACTGCCCATGCCTTCGTAATAATTTTCTTTATAGTAATACCAATTATGATTGGAGGCT 174**

**JF292411 TCACTGCCCATGCCTTCGTAATAATTTTCTTTATAGTAATACCAATTATGATTGGAGGCT 174**

*************************************************** ************

**PX795198 TTGGAAACTGACTTGTCCCCTTAATAATTGGAGCGCCTGATATGGCATTCCCTCGAATAA 223**

**PX795199 TTGGAAACTGACTTGTCCCCTTAATAATTGGAGCGCCTGATATGGCATTCCCTCGAATAA 223**

**PX312189 TTGGAAACTGACTTGTCCCCTTAATAATTGGAGCGCCTGATATGGCATTCCCTCGAATAA 223**

**PX795204 TTGGAAACTGACTTGTCCCCTTAATAATTGGAGCGCCTGATATGGCATTCCCTCGAATAA 225**

**MW829646 TTGGAAACTGACTTGTCCCCTTAATAATTGGAGCGCCTGATATGGCATTCCCTCGAATAA 225**

**PX795205 TTGGAAACTGACTTGTCCCCTTAATAATTGGAGCGCCTGATATGGCATTCCCTCGAATAA 226**

**PX795200 TTGGAAACTGACTTGTCCCCTTAATAATTGGAGCGCCTGATATGGCATTCCCTCGAATAA 223**

**PX795201 TTGGAAACTGACTTGTCCCCTTAATAATTGGAGCGCCTGATATGGCATTCCCTCGAATAA 237**

**PX795202 TTGGAAACTGACTTGTCCCCTTAATAATTGGAGCGCCTGATATGGCATTCCCTCGAATAA 223**

**PX795203 TTGGAAACTGACTTGTCCCCTTAATAATTGGAGCGCCTGATATGGCATTCCCTCGAATAA 237**

**JF292393 TTGGAAACTGACTTGTCCCCTTAATAATTGGAGCGCCTGATATGGCATTCCCTCGAATAA 223**

**PP396129 TTGGAAACTGACTTGTCCCCTTAATAATTGGAGCGCCTGATATGGCATTCCCTCGAATAA 237**

**PX795197 TCGGAAACTGACTTGTCCCCTTAATAATTGGAGCACCAGATATGGCATTTCCACGAATAA 223**

**JF292425 TCGGAAACTGACTTGTCCCCTTAATAATTGGAGCACCAGATATGGCATTTCCACGAATAA 223**

**PX795196 TCGGAAACTGACTTGTCCCCTTAATAATTGGAGCACCAGATATGGCATTTCCACGAATAA 240**

**JF292419 TCGGAAACTGACTTGTCCCCTTAATAATTGGAGCACCAGATATGGCATTTCCACGAATAA 240**

**PX795194 TCGGAAACTGACTTGTCCCCTTAATAATTGGGGCACCAGATATGGCATTTCCACGAATAA 231**

**JF292415 TCGGAAACTGACTTGTCCCCTTAATAATTGGGGCACCAGATATGGCATTTCCACGAATAA 231**

**PX795195 TCGGAAACTGACTTGTCCCCTTAATAATTGGAGCACCAGATATGGCATTTCCACGAATAA 234**

**JF292411 TCGGAAACTGACTTGTCCCCTTAATAATTGGAGCACCAGATATGGCATTTCCACGAATAA 234**

*** ***************************** ** ** *********** ** *********

**PX795198 ATAATATGAGTTTTTGATTACTTCCGCCTTCCTTCCTACTATTGCTTGCCTCCTCTGGAG 283**

**PX795199 ATAATATGAGTTTTTGATTACTTCCGCCTTCCTTCCTACTATTGCTTGCCTCCTCTGGAG 283**

**PX312189 ATAATATGAGTTTTTGATTACTTCCGCCTTCCTTCCTACTATTGCTTGCCTCCTCTGGAG 283**

**PX795204 ATAATATGAGTTTTTGATTACTTCCGCCTTCCTTCCTACTATTGCTTGCCTCCTCTGGAG 285**

**MW829646 ATAATATGAGTTTTTGATTACTTCCGCCTTCCTTCCTACTATTGCTTGCCTCCTCTGGAG 285**

**PX795205 ATAATATGAGTTTTTGATTACTTCCGCCTTCCTTCCTACTATTGCTTGCCTCCTCTGGAG 286**

**PX795200 ATAATATGAGTTTTTGATTACTTCCGCCTTCCTTCCTACTATTGCTTGCCTCCTCTGGAG 283**

**PX795201 ATAATATGAGTTTTTGATTACTTCCGCCTTCCTTCCTACTATTGCTTGCCTCCTCTGGAG 297**

**PX795202 ATAATATGAGTTTTTGATTACTTCCGCCTTCCTTCCTACTATTGCTTGCCTCCTCTGGAG 283**

**PX795203 ATAATATGAGTTTTTGATTACTTCCGCCTTCCTTCCTACTATTGCTTGCCTCCTCTGGAG 297**

**JF292393 ATAATATGAGTTTTTGATTACTTCCGCCTTCCTTCCTACTATTGCTTGCCTCCTCTGGAG 283**

**PP396129 ATAATATGAGTTTTTGATTACTTCCGCCTTCCTTCCTACTATTGCTTGCCTCCTCTGGAG 297**

**PX795197 ATAACATGAGTTTTTGATTACTCCCACCTTCCTTCCTACTACTGCTCGCCTCATCTGGAG 283**

**JF292425 ATAACATGAGTTTTTGATTACTCCCACCTTCCTTCCTACTACTGCTCGCCTCATCTGGAG 283**

**PX795196 ATAACATGAGTTTTTGATTACTCCCACCTTCCTTCCTACTACTGCTCGCCTCATCTGGAG 300**

**JF292419 ATAACATGAGTTTTTGATTACTCCCACCTTCCTTCCTACTACTGCTCGCCTCATCTGGAG 300**

**PX795194 ATAACATGAGTTTTTGATTACTCCCACCTTCCTTCCTACTACTGCTCGCCTCATCTGGAG 291**

**JF292415 ATAACATGAGTTTTTGATTACTCCCACCTTCCTTCCTACTACTGCTCGCCTCATCTGGAG 291**

**PX795195 ATAACATGAGTTTTTGATTACTCCCACCTTCCTTCCTACTACTGCTCGCCTCATCTGGAG 294**

**JF292411 ATAACATGAGTTTTTGATTACTCCCACCTTCCTTCCTACTACTGCTCGCCTCATCTGGAG 294**

****** ***************** ** *************** **** ***** *********

**PX795198 TAGAAGCAGGGGCAGGAACAGGATGAACTGTATATCCACCCCTTGCTGGAAACCTCGCAC 343**

**PX795199 TAGAAGCAGGGGCAGGAACAGGATGAACTGTATATCCACCCCTTGCTGGAAACCTCGCAC 343**

**PX312189 TAGAAGCAGGGGCAGGAACAGGATGAACTGTATATCCACCCCTTGCTGGAAACCTCGCAC 343**

**PX795204 TAGAAGCAGGGGCAGGAACAGGATGAACTGTATATCCACCCCTTGCTGGAAACCTCGCAC 345**

**MW829646 TAGAAGCAGGGGCAGGAACAGGATGAACTGTATATCCACCCCTTGCTGGAAACCTCGCAC 345**

**PX795205 TAGAAGCAGGGGCAGGAACAGGATGAACTGTATATCCACCCCTTGCTGGAAACCTCGCAC 346**

**PX795200 TAGAAGCAGGGGCAGGAACAGGATGAACTGTATATCCACCCCTTGCTGGAAACCTCGCAC 343**

**PX795201 TAGAAGCAGGGGCAGGAACAGGATGAACTGTATATCCACCCCTTGCTGGAAACCTCGCAC 357**

**PX795202 TAGAAGCAGGGGCAGGAACAGGATGAACTGTATATCCACCCCTTGCTGGAAACCTCGCAC 343**

**PX795203 TAGAAGCAGGGGCAGGAACAGGATGAACTGTATATCCACCCCTTGCTGGAAACCTCGCAC 357**

**JF292393 TAGAAGCAGGGGCAGGAACAGGATGAACTGTATATCCACCCCTTGCTGGAAACCTCGCAC 343**

**PP396129 TAGAAGCAGGGGCAGGAACAGGATGAACTGTATATCCACCCCTTGCTGGAAACCTCGCAC 357**

**PX795197 TTGAAGCGGGGGCAGGAACAGGATGAACTGTCTACCCACCTTTAGCCGGAAACCTAGCAC 343**

**JF292425 TTGAAGCGGGGGCAGGAACAGGATGAACTGTCTACCCACCTTTAGCCGGAAACCTAGCAC 343**

**PX795196 TTGAAGCGGGGGCAGGAACAGGATGAACTGTCTACCCACCTTTAGCCGGAAACCTAGCAC 360**

**JF292419 TTGAAGCGGGGGCAGGAACAGGATGAACTGTCTACCCACCTTTAGCCGGAAACCTAGCAC 360**

**PX795194 TTGAAGCGGGGGCAGGAACAGGATGAACTGTCTACCCACCTTTAGCCGGAAACCTAGCAC 351**

**JF292415 TTGAAGCGGGGGCAGGAACAGGATGAACTGTCTACCCACCTTTAGCCGGAAACCTAGCAC 351**

**PX795195 TTGAAGCGGGGGCAGGAACAGGATGAACTGTCTACCCACCTTTAGCCGGAAACCTAGCAC 354**

**JF292411 TTGAAGCGGGGGCAGGAACAGGATGAACTGTCTACCCACCTTTAGCCGGAAACCTAGCAC 354**

*** ***** *********************** ** ***** * ** ******** ******

**PX795198 ATGCCGGGGCTTCTGTAGATTTAACTATTTTCTCCCTTCATCTTGCAGGGGTATCATCCA 403**

**PX795199 ATGCCGGGGCTTCTGTAGATTTAACTATTTTCTCCCTTCATCTTGCAGGGGTATCATCCA 403**

**PX312189 ATGCCGGGGCTTCTGTAGATTTAACTATTTTCTCCCTTCATCTTGCAGGGGTATCATCCA 403**

**PX795204 ATGCCGGGGCTTCTGTAGATTTAACTATTTTCTCCCTTCATCTTGCAGGGGTATCATCCA 405**

**MW829646 ATGCCGGGGCTTCTGTAGATTTAACTATTTTCTCCCTTCATCTTGCAGGGGTATCATCCA 405**

**PX795205 ATGCCGGGGCTTCTGTAGATTTAACTATTTTCTCCCTTCATCTTGCAGGGGTATCATCCA 406**

**PX795200 ATGCCGGGGCTTCTGTAGATTTAACTATTTTCTCCCTTCATCTTGCAGGGGTATCATCCA 403**

**PX795201 ATGCCGGGGCTTCTGTAGATTTAACTATTTTCTCCCTTCATCTTGCAGGGGTATCATCCA 417**

**PX795202 ATGCCGGGGCTTCTGTAGATTTAACTATTTTCTCCCTTCATCTTGCAGGGGTATCATCCA 403**

**PX795203 ATGCCGGGGCTTCTGTAGATTTAACTATTTTCTCCCTTCATCTTGCAGGGGTATCATCCA 417**

**JF292393 ATGCCGGGGCTTCTGTAGATTTAACTATTTTCTCCCTTCATCTTGCAGGGGTATCATCCA 403**

**PP396129 ATGCCGGGGCTTCTGTAGATTTAACTATTTTCTCCCTTCATCTTGCAGGGGTATCATCCA 417**

**PX795197 ATGCCGGAGCTTCTGTAGATTTAACTATTTTCTCTCTTCACTTGGCAGGGGTATCATCTA 403**

**JF292425 ATGCCGGAGCTTCTGTAGATTTAACTATTTTCTCTCTTCACTTGGCAGGGGTATCATCTA 403**

**PX795196 ATGCCGGAGCTTCTGTAGATTTAACTATTTTCTCTCTTCACTTGGCAGGGGTATCATCTA 420**

**JF292419 ATGCCGGAGCTTCTGTAGATTTAACTATTTTCTCTCTTCACTTGGCAGGGGTATCATCTA 420**

**PX795194 ATGCCGGAGCTTCTGTAGATTTAACTATTTTCTCTCTTCACTTGGCAGGGGTATCATCTA 411**

**JF292415 ATGCCGGAGCTTCTGTAGATTTAACTATTTTCTCTCTTCACTTGGCAGGGGTATCATCTA 411**

**PX795195 ATGCCGGAGCTTCTGTAGATTTAACTATTTTCTCTCTTCACTTGGCAGGGGTATCATCTA 414**

**JF292411 ATGCCGGAGCTTCTGTAGATTTAACTATTTTCTCTCTTCACTTGGCAGGGGTATCATCTA 414**

********* ************************** ***** * ************** ***

**PX795198 TTCTAGGAGCCATTAATTTTATTACAACCATTATTAACATAAAACCACCAGCAATTTCAC 463**

**PX795199 TTCTAGGAGCCATTAATTTTATTACAACCATTATTAACATAAAACCACCAGCAATTTCAC 463**

**PX312189 TTCTAGGAGCCATTAATTTTATTACAACCATTATTAACATAAAACCACCAGCAATTTCAC 463**

**PX795204 TTCTAGGAGCCATTAATTTTATTACAACCATTATTAACATAAAACCACCAGCAATTTCAC 465**

**MW829646 TTCTAGGAGCCATTAATTTTATTACAACCATTATTAACATAAAACCACCAGCAATTTCAC 465**

**PX795205 TTCTAGGAGCCATTAATTTTATTACAACCATTATTAACATAAAACCACCAGCAATTTCAC 466**

**PX795200 TTCTAGGAGCCATTAATTTTATTACAACCATTATTAACATAAAACCACCAGCAATTTCAC 463**

**PX795201 TTCTAGGAGCCATTAATTTTATTACAACCATTATTAACATAAAACCACCAGCAATTTCAC 477**

**PX795202 TTCTAGGAGCCATTAATTTTATTACAACCATTATTAACATAAAACCACCAGCAATTTCAC 463**

**PX795203 TTCTAGGAGCCATTAATTTTATTACAACCATTATTAACATAAAACCACCAGCAATTTCAC 477**

**JF292393 TTCTAGGAGCCATTAATTTTATTACAACCATTATTAACATAAAACCACCAGCAATTTCAC 463**

**PP396129 TTCTAGGAGCCATTAATTTTATTACAACCATTATTAACATAAAACCACCAGCAATTTCAC 477**

**PX795197 TTCTAGGGGCCATTAACTTTATCACAACTATTATTAATATAAAACCCCCAGCAATTTCAC 463**

**JF292425 TTCTAGGGGCCATTAACTTTATCACAACTATTATTAATATAAAACCCCCAGCAATTTCAC 463**

**PX795196 TTCTAGGGGCCATTAACTTTATCACAACTATTATTAATATAAAACCCCCAGCAATTTCAC 480**

**JF292419 TTCTAGGGGCCATTAACTTTATCACAACTATTATTAATATAAAACCCCCAGCAATTTCAC 480**

**PX795194 TTCTAGGGGCCATTAACTTTATCACAACTATTATTAATATAAAACCCCCAGCAATTTCAC 471**

**JF292415 TTCTAGGGGCCATTAACTTTATCACAACTATTATTAATATAAAACCCCCAGCAATTTCAC 471**

**PX795195 TTCTAGGGGCCATTAACTTTATCACAACTATTATTAATATAAAACCCCCAGCAATTTCAC 474**

**JF292411 TTCTAGGGGCCATTAACTTTATCACAACTATTATTAATATAAAACCCCCAGCAATTTCAC 474**

********* ******** ***** ***** ******** ******** ***************

**PX795198 AATATCAAACACCTTTATTTGTATGGGCTGTCTTAATTACAGCTGTTCTTCTATTATTAT 523**

**PX795199 AATATCAAACACCTTTATTTGTATGGGCTGTCTTAATTACAGCTGTTCTTCTATTATTAT 523**

**PX312189 AATATCAAACACCTTTATTTGTATGGGCTGTCTTAATTACAGCTGTTCTTCTATTATTAT 523**

**PX795204 AATATCAAACACCTTTATTTGTATGGGCCGTCTTAATTACAGCTGTTCTTCTATTATTAT 525**

**MW829646 AATATCAAACACCTTTATTTGTATGGGCCGTCTTAATTACAGCTGTTCTTCTATTATTAT 525**

**PX795205 AATATCAAACACCTTTATTTGTATGGGCTGTCTTAATTACAGCTGTTCTTCTATTATTAT 526**

**PX795200 AATATCAAACACCTTTATTTGTATGGGCTGTCTTAATTACAGCTGTTCTTCTATTATTAT 523**

**PX795201 AATATCAAACACCTTTATTTGTATGGGCTGTCTTAATTACAGCTGTTCTTCTATTATTAT 537**

**PX795202 AATATCAAACACCTTTATTTGTATGGGCTGTCTTAATTACAGCTGTTCTTCTATTATTAT 523**

**PX795203 AATATCAAACACCTTTATTTGTATGGGCTGTCTTAATTACAGCTGTTCTTCTATTATTAT 537**

**JF292393 AATATCAAACACCTTTATTTGTATGGGCTGTCTTAATTACAGCTGTTCTTCTATTATTAT 523**

**PP396129 AATATCAAACACCTTTATTTGTATGGGCTGTCTTAATTACAGCTGTTCTTCTATTATTAT 537**

**PX795197 AGTACCAAACACCTCTATTTGTATGAGCTGTACTAATTACAGCTGTACTTCTACTGCTAT 523**

**JF292425 AGTACCAAACACCTCTATTTGTATGAGCTGTACTAATTACAGCTGTACTTCTACTGCTAT 523**

**PX795196 AGTACCAAACACCTCTATTTGTATGAGCTGTACTAATTACAGCTGTACTTCTACTGCTAT 540**

**JF292419 AGTACCAAACACCTCTATTTGTATGAGCTGTACTAATTACAGCTGTACTTCTACTGCTAT 540**

**PX795194 AGTACCAAACACCTCTATTTGTATGAGCTGTACTAATTACAGCTGTACTTCTACTGCTAT 531**

**JF292415 AGTACCAAACACCTCTATTTGTATGAGCTGTACTAATTACAGCTGTACTTCTACTGCTAT 531**

**PX795195 AGTACCAAACACCTCTATTTGTATGAGCTGTACTAATTACAGCTGTACTTCTACTGCTAT 534**

**JF292411 AGTACCAAACACCTCTATTTGTATGAGCTGTACTAATTACAGCTGTACTTCTACTGCTAT 534**

*** ** ********* ********** ** ** ************* ****** * *****

**PX795198 CTCTACCAGTACTGGCTGCCGGCATTACTATACTCCTAACAGATCGAAACCTAAATACTA 583**

**PX795199 CTCTACCAGTACTGGCTGCCGGCATTACTATACTCCTAACAGATCGAAACCTAAATACTA 583**

**PX312189 CTCTACCAGTACTGGCTGCCGGCATTACTATACTCCTAACAGATCGAAACCTAAATACTA 583**

**PX795204 CTCTACCAGTACTGGCTGCCGGCATTACTATACTCCTAACAGATCGAAACCTAAATACTA 585**

**MW829646 CTCTACCAGTACTGGCTGCCGGCATTACTATACTCCTAACAGATCGAAACCTAAATACTA 585**

**PX795205 CTCTACCAGTACTGGCTGCCGGCATTACTATACTCCTAACAGATCGAAACCTAAATACTA 586**

**PX795200 CTCTACCAGTACTGGCTGCCGGCATTACTATACTCCTAACAGATCGAAACCTAAATACTA 583**

**PX795201 CTCTACCAGTACTGGCTGCCGGCATTACTATACTCCTAACAGATCGAAACCTAAATACTA 597**

**PX795202 CTCTACCAGTACTGGCTGCCGGCATTACTATACTCCTAACAGATCGAAACCTAAATACTA 583**

**PX795203 CTCTACCAGTACTGGCTGCCGGCATTACTATACTCCTAACAGATCGAAACCTAAATACTA 597**

**JF292393 CTCTACCAGTACTGGCTGCCGGCATTACTATACTCCTAACAGATCGAAACCTAAATACTA 583**

**PP396129 CTCTACCAGTACTGGCTGCCGGCATTACTATACTCCTAACAGATCGAAACCTAAATACTA 597**

**PX795197 CTCTGCCCGTACTAGCCGCCGGCATTACAATACTTCTAACAGACCGAAATCTAAATACTA 583**

**JF292425 CTCTGCCCGTACTAGCCGCCGGCATTACAATACTTCTAACAGACCGAAATCTAAATACTA 583**

**PX795196 CTCTGCCCGTACTAGCCGCCGGCATTACAATACTTCTAACAGACCGAAATCTAAATACTA 600**

**JF292419 CTCTGCCCGTACTAGCCGCCGGCATTACAATACTTCTAACAGACCGAAATCTAAATACTA 600**

**PX795194 CTCTGCCCGTACTAGCCGCCGGCATTACAATACTTCTAACAGACCGAAATCTAAATACTA 591**

**JF292415 CTCTGCCCGTACTAGCCGCCGGCATTACAATACTTCTAACAGACCGAAATCTAAATACTA 591**

**PX795195 CTCTGCCCGTACTAGCCGCCGGCATTACAATACTTCTAACAGACCGAAATCTAAATACTA 594**

**JF292411 CTCTGCCCGTACTAGCCGCCGGCATTACAATACTTCTAACAGACCGAAATCTAAATACTA 594**

****** ** ***** ** *********** ***** ******** ***** ************

**PX795198 CATTCTTTGATCCTGCAGGCGGAGGAGATCCTATTTTATATCAACATCTTTTCTGATTCT 643**

**PX795199 CATTCTTTGATCCTGCAGGCGGAGGAGATCCTATTTTATATCAACATCTTTTCTGATTCT 643**

**PX312189 CATTCTTTGATCCTGCAGGCGGAGGAGATCCTATTTTATATCAACATCTTTTCTGATTCT 643**

**PX795204 CATTCTTTGATCCTGCAGGCGGAGGAGATCCTATTTTATATCAACATCTTTTCTGATTCT 645**

**MW829646 CATTCTTTGATCCTGCAGGCGGAGGAGATCCTATTTTATATCAACATCTTTTCTGATTCT 645**

**PX795205 CATTCTTTGATCCTGCAGGCGGAGGAGATCCTATTTTATATCAACATCTTTTCTGATTCT 646**

**PX795200 CATTCTTTGATCCTGCAGGCGGAGGAGATCCTATTTTATATCAACATCTTTTCTGATTCT 643**

**PX795201 CATTCTTTGATCCTGCAGGCGGAGGAGATCCTATTTTATATCAACATCTTTTCTGATTCT 657**

**PX795202 CATTCTTTGATCCTGCAGGCGGAGGAGATCCTATTTTATATCAACATCTTTTCTGATTCT 643**

**PX795203 CATTCTTTGATCCTGCAGGCGGAGGAGATCCTATTTTATATCAACATCTTTTCTGATTCT 657**

**JF292393 CATTCTTTGATCCTGCAGGCGGAGGAGATCCTATTTTATATCAACATCTTTTCTGATTCT 643**

**PP396129 CATTCTTTGATCCTGCAGGCGGAGGAGATCCTATTTTATATCAACATCTTTTCTGATTCT 657**

**PX795197 CATTCTTCGACCCCGCAGGGGGAGGAGACCCCATCCTATATCAACAGCTTTTCTGATTCT 643**

**JF292425 CATTCTTCGACCCCGCAGGGGGAGGAGACCCCATCCTATATCAACACCTTTTCTGATTCT 643**

**PX795196 CATTCTTCGACCCCGCAGGGGGAGGAGACCCCATCCTATATCAACACCTTT--------- 651**

**JF292419 CATTCTTCGACCCCGCAGGGGGAGGAGACCCCATCCTATATCAACACCTTT--------- 651**

**PX795194 CATTCTTCGACCCCGCAGGGGGAGGAGACCCCATCCTATATCAACACCTTTTCTGATTCT 651**

**JF292415 CATTCTTCGACCCCGCAGGGGGAGGAGACCCCATCCTATATCAACACCTTTTCTGATTCT 651**

**PX795195 CATTCTTCGACCCCGCAGGGGGAGGAGACCCCATCCTATATCAACACCTTTTCTGAT--- 651**

**JF292411 CATTCTTCGACCCCGCAGGGGGAGGAGACCCCATCCTATATCAACACCTTTTCTGAT--- 651**

********* ** ** ***** ******** ** ** ********** ******

**PX795198 TCCGTCAC--- 651**

**PX795199 TCCGTCAC--- 651**

**PX312189 TCCGTCAC--- 651**

**PX795204 TCGGTCACCC- 655**

**MW829646 TCGGTCACCC- 655**

**PX795205 TCGGT------ 651**

**PX795200 TCGGTCAC--- 651**

**PX795201 TCGGTCACCCT 668**

**PX795202 TCGGTCAC--- 651**

**PX795203 TCGGTCACCCT 668**

**JF292393 TCGGTCAC--- 651**

**PP396129 TCGGTCACCCT 668**

**PX795197 TCGGTCAC--- 651**

**JF292425 TCGGTCAC--- 651**

**PX795196 ----------- 651**

**JF292419 ----------- 651**

**PX795194 ----------- 651**

**JF292415 ----------- 651**

**PX795195 ----------- 651**

**JF292411 ----------- 651**

**Figure S2.** Multiple sequence alignment of imported fish fillets in Egyptian markets against reference sequences from GenBank, generated by Clustal Omega. Identities and similarities are indicated by star (*) and gaps are indicated by dashes (-).

**KJ590087 -------------------------------------GCCTGCCCAGTGACTTCAAGTTC 23**

**JF292297 -----------------------------------GTGCCTGGGCCGGTATAGTCGGCAC 25**

**JF292324 -------------------CTTTATCTAGTATTTGGTGCCTGAGCCGGAATAGTCGGCAC 41**

**JF292311 --------------------TTTATCTAGTATTTGGTGCCTGATCCGGAATAGTCGGCAC 40**

**JF292339 CCATAAAGACATTGGCACCCTCTACCTAGTGTTCGGTGCCTGAGCTGGAATAGTTGGCAC 60**

**PX795198 -------------------------------------GCCTGAGCAGGAATAGTTGGTAC 23**

**PX795199 -------------------------------------GCCTGAGCAGGAATAGTTGGTAC 23**

**PX795204 -----------------------------------GTGCCTGAGCAGGAATAGTTGGTAC 25**

**PX795205 ----------------------------------GGTGCCTGAGCAGGAATAGTTGGTAC 26**

**PX795200 -------------------------------------GCCTGAGCAGGAATAGTTGGTAC 23**

**PX795201 -----------------------ACCTAGTATTTGGTGCCTGAGCAGGAATAGTTGGTAC 37**

**PX795202 -------------------------------------GCCTGAGCAGGAATAGTTGGTAC 23**

**PX795203 -----------------------ACCTAGTATTTGGTGCCTGAGCAGGAATAGTTGGTAC 37**

**JF292393 -------------------------------------GCCTGAGCAGGAATAGTTGGTAC 23**

**PX795197 -------------------------------------GCCTGAGCCGGAATAGTTGGTAC 23**

**PX795196 --------------------TCTATTTAGTGTTTGGTGCCTGAGCCGGAATAGTTGGTAC 40**

**PX795194 -----------------------------TGTTTGGTGCCTGAGCCGGAATAGTTGGTAC 31**

**JF292415 -----------------------------TGTTTGGTGCCTGAGCCGGAATAGTTGGTAC 31**

**PX795195 --------------------------TAGTGTTTGGTGCCTGAGCCGGAATAGTTGGTAC 34**

**JF292338 ---------------------CCTTTGGCTTTTTGGTGCCTGAGCCGGAATAGTTGGCAC 39**

**JF292368 -----------------------------------GTGCCTGAGCCGGAATAGTCGGCAC 25**

**JF292353 -----------------------------------------GAGCCGGAATAGTGGGTAC 19**

*** * * * * ***

**KJ590087 AACGGCCGCGGTATTCTGACCGTGCAAAGGTAGCGCAATCACTCGTCCCTTAAATAA--- 80**

**JF292297 AGC-CCTAAGCTTACTAATCCGGGCGGAACTGGCACAACCAGGGGCTCTTTTAGGAGATG 84**

**JF292324 AGC-CCTAAGCCTACTAATCCGAGCAGAACTGGCACAGCCTGGGGCTCTTCTAGGAGATG 100**

**JF292311 AGC-CCTTAGCTTATTAATCCGGGCGGAACTAGCACAACCCGGAGCCCTTTTAGGAGACG 99**

**JF292339 AGC-TCTTAGCTTACTAATTCGAGCAGAGCTAGCCCAACCCGGCGCCCTTCTAGGCGATG 119**

**PX795198 GGC-CCTCAGCCTCCTAATTCGGGCAGAGCTAGCCCAACCCGGCGCCCTTCTAGGCGACG 82**

**PX795199 GGC-CCTCAGCCTCCTAATTCGGGCAGAGCTAGCCCAACCCGGCGCCCTTCTAGGCGACG 82**

**PX795204 GGC-CCTCAGCCTCCTAATTCGGGCAGAGCTAGCCCAACCCGGCGCCCTTCTAGGCGACG 84**

**PX795205 GGC-CCTCAGCCTCCTAATTCGGGCAGAGCTAGCCCAACCCGGCGCCCTTCTAGGCGACG 85**

**PX795200 GGC-CCTCAGCCTCCTAATTCGGGCAGAGCTAGCCCAACCCGGCGCCCTTCTAGGCGACG 82**

**PX795201 GGC-CCTCAGCCTCCTAATTCGGGCAGAGCTAGCCCAACCCGGCGCCCTTCTAGGCGACG 96**

**PX795202 GGC-CCTCAGCCTCCTAATTCGGGCAGAGCTAGCCCAACCCGGCGCCCTTCTAGGCGACG 82**

**PX795203 GGC-CCTCAGCCTCCTAATTCGGGCAGAGCTAGCCCAACCCGGCGCCCTTCTAGGCGACG 96**

**JF292393 GGC-CCTCAGCCTCCTAATTCGGGCAGAGCTAGCCCAACCCGGCGCCCTTCTAGGCGACG 82**

**PX795197 AGC-CCTTAGCCTGCTAATTCGGGCAGAGCTAGCCCAGCCCGGCGCCCTTCTGGGCGACG 82**

**PX795196 AGC-CCTTAGCCTCCTAATTCGGGCAGAGCTAGCCCAGCCCGGCGCCCTTCTGGGCGACG 99**

**PX795194 AGC-CCTTAGCCTGCTAATTCGGGCAGAGCTAGCCCAGCCCGGCGCCCTTCTGGGCGACG 90**

**JF292415 AGC-CCTTAGCCTGCTAATTCGGGCAGAGCTAGCCCAGCCCGGCGCCCTTCTGGGCGACG 90**

**PX795195 AGC-CCTTAGCCTGCTAATTCCGGCAGAGCTAGCCCAGCCCGGCGCCCTTCTGGGCGACG 93**

**JF292338 AGC-TCTTAGCCTACTAATTCGAGCAGAGCTAGCCCAACCTGGGGCCCTCCTAGGTGATG 98**

**JF292368 GGC-CCTTAGCCTGCTTATCCGGGCAGAGCTAGCCCAGCCCGGTGCCCTTCTAGGCGATG 84**

**JF292353 CGC-CCTTAGCCTGCTTATCCGGGCGGAATTAGCCCAACCCGGCGCCCTTCTAGGCGATG 78**

*** * * * ** * * ** ** * * ***

**KJ590087 ------GGTCCTGTATGAATGG-------CTAGACGAGGGCTTAACTGTCTCC------- 120**

**JF292297 ACCAAATTTACAATGTTATTGTTACTGCCCACGCCTTCGTAATAATTTTCTTTATAGTAA 144**

**JF292324 ACCAGATCTATAATGTTATTGTCACCGCCCACGCCTTCGTAATAATCTTCTTTATAGTAA 160**

**JF292311 ACCAGATTTATAATGTTATTGTTACTGCACACGCCTTCGTAATAATCTTCTTTATAGTAA 159**

**JF292339 ATCAAATTTACAATGTTATTGTTACTGCTCACGCCTTCATTATGATTTTCTTTATAGTAA 179**

**PX795198 ACCAAATTTATAATGTTATTGTCACTGCCCATGCCTTCGTAATAATTTTCTTTATAGTAA 142**

**PX795199 ACCAAATTTATAATGTTATTGTCACTGCCCATGCCTTCGTAATAATTTTCTTTATAGTAA 142**

**PX795204 ACCAAATTTATAATGTTATTGTCACTGCCCATGCCTTCGTAATAATTTTCTTTATAGTAA 144**

**PX795205 ACCAAATTTATAATGTTATTGTCACTGCCCATGCCTTCGTAATAATTTTCTTTATAGTAA 145**

**PX795200 ACCAAATTTATAATGTTATTGTCACTGCCCATGCCTTCGTAATAATTTTCTTTATAGTAA 142**

**PX795201 ACCAAATTTATAATGTTATTGTCACTGCCCATGCCTTCGTAATAATTTTCTTTATAGTAA 156**

**PX795202 ACCAAATTTATAATGTTATTGTCACTGCCCATGCCTTCGTAATAATTTTCTTTATAGTAA 142**

**PX795203 ACCAAATTTATAATGTTATTGTCACTGCCCATGCCTTCGTAATAATTTTCTTTATAGTAA 156**

**JF292393 ACCAAATTTATAATGTTATTGTCACTGCCCATGCCTTCGTAATAATTTTCTTTATAGTAA 142**

**PX795197 ACCAAATTTATAATGTCATTGTCACTGCCCATGCCTTCGTAATAATTTTCTTTATAGTAA 142**

**PX795196 ACCAAATTTATAATGTCATTGTCACTGCCCATGCCTTCGTAATAATTTTCTTTATAGTAA 159**

**PX795194 ACCAAATTTATAATGTCATTGTCACTGCCCATGCCTTCGTAATAATTTTCTTTATAGTAA 150**

**JF292415 ACCAAATTTATAATGTCATTGTCACTGCCCATGCCTTCGTAATAATTTTCTTTATAGTAA 150**

**PX795195 ACCAAATTTATAATGTCATTGTCACTGCCCATGCCTTCGTAATAATTTTCTTTATAGTAA 153**

**JF292338 ATCAGATTTACAATGTTATTGTTACTGCTCATGCCTTCGTAATAATCTTCTTCATAGTAA 158**

**JF292368 ACCAAATTTATAATGTTATTGTCACTGCCCACGCCTTTGTAATAATTTTCTTTATAGTAA 144**

**JF292353 ACCAAATTTACAATGTTATTGTTACTGCTCACGCCTTTGTAATAATTTTCTTTATAGTAA 138**

*** * * * ** * * * * * *****

**KJ590087 ---CCCTTCAGGTCAGTG-------AAATTGATCTA---------TCCGTGCAGAAGCGG 161**

**JF292297 TACCAATTATAATCGGGGGTTTTGGAAACTGACTTGTACCCCTAATAATTGGGGCACCCG 204**

**JF292324 TACCAATTATGATGGGGGGCTTCGGAAACTGACTTGTGCCCCTAATAATTGGTGCCCCCG 220**

**JF292311 TACCAATTATGATTGGTGGCTTCGGAAACTGACTAGTACCACTAATGCTGGGGGCCCCTG 219**

**JF292339 TACCAATTATAATTGGAGGCTTTGGGAATTGACTTGTACCGCTGATAATTGGAGCACCAG 239**

**PX795198 TACCAATTATAATTGGAGGCTTTGGAAACTGACTTGTCCCCTTAATAATTGGAGCGCCTG 202**

**PX795199 TACCAATTATAATTGGAGGCTTTGGAAACTGACTTGTCCCCTTAATAATTGGAGCGCCTG 202**

**PX795204 TACCAATTATAATTGGAGGCTTTGGAAACTGACTTGTCCCCTTAATAATTGGAGCGCCTG 204**

**PX795205 TACCAATTATAATTGGAGGCTTTGGAAACTGACTTGTCCCCTTAATAATTGGAGCGCCTG 205**

**PX795200 TACCAATTATAATTGGAGGCTTTGGAAACTGACTTGTCCCCTTAATAATTGGAGCGCCTG 202**

**PX795201 TACCAATTATAATTGGAGGCTTTGGAAACTGACTTGTCCCCTTAATAATTGGAGCGCCTG 216**

**PX795202 TACCAATTATAATTGGAGGCTTTGGAAACTGACTTGTCCCCTTAATAATTGGAGCGCCTG 202**

**PX795203 TACCAATTATAATTGGAGGCTTTGGAAACTGACTTGTCCCCTTAATAATTGGAGCGCCTG 216**

**JF292393 TACCAATTATAATTGGAGGCTTTGGAAACTGACTTGTCCCCTTAATAATTGGAGCGCCTG 202**

**PX795197 TACCAATTATGATTGGAGGCTTCGGAAACTGACTTGTCCCCTTAATAATTGGAGCACCAG 202**

**PX795196 TACCAATTATGATTGGAGGCTTCGGAAACTGACTTGTCCCCTTAATAATTGGAGCACCAG 219**

**PX795194 TACCAATTATGATTGGAGGCTTCGGAAACTGACTTGTCCCCTTAATAATTGGGGCACCAG 210**

**JF292415 TACCAATTATGATTGGAGGCTTCGGAAACTGACTTGTCCCCTTAATAATTGGGGCACCAG 210**

**PX795195 TACCAATTATGATTGGAGGCTTCGGAAACTGACTTGTCCCCTTAATAATTGGAGCACCAG 213**

**JF292338 TACCAATTATAATTGGGGGCTTTGGAAACTGACTTGTTCCACTAATAATTGGGGCACCCG 218**

**JF292368 TGCCAATTATGATTGGAGGGTTTGGAAATTGGCTTGTTCCCCTAATGATTGGGGCACCAG 204**

**JF292353 TACCAATTATGATTGGAGGGTTTGGAAACTGGCTTGTTCCCCTAATGATCGGAGCGCCAG 198**

*** ** * * * ** ** * * * * ***

**KJ590087 ATATAAGAATACAAGACGAGAAGACCCTTTGGAG---CTTTAGGTACAAGGCTTACTTAC 218**

**JF292297 ATATAGCATTCCCACGAATGAATAACATAAGCTTCTGATTACTGCCCCCATCTTTTCTAC 264**

**JF292324 ATATAGCATTCCCACGAATAAATAACATAAGCTTTTGACTACTCCCACCATCATTCCTAC 280**

**JF292311 ATATAGCATTCCCACGAATAAATAATATAAGCTTCTGACTACTACCCCCATCATTCCTAT 279**

**JF292339 ACATAGCATTCCCCCGAATAAACAACATAAGCTTCTGATTATTGCCCCCCTCCTTTCTAT 299**

**PX795198 ATATGGCATTCCCTCGAATAAATAATATGAGTTTTTGATTACTTCCGCCTTCCTTCCTAC 262**

**PX795199 ATATGGCATTCCCTCGAATAAATAATATGAGTTTTTGATTACTTCCGCCTTCCTTCCTAC 262**

**PX795204 ATATGGCATTCCCTCGAATAAATAATATGAGTTTTTGATTACTTCCGCCTTCCTTCCTAC 264**

**PX795205 ATATGGCATTCCCTCGAATAAATAATATGAGTTTTTGATTACTTCCGCCTTCCTTCCTAC 265**

**PX795200 ATATGGCATTCCCTCGAATAAATAATATGAGTTTTTGATTACTTCCGCCTTCCTTCCTAC 262**

**PX795201 ATATGGCATTCCCTCGAATAAATAATATGAGTTTTTGATTACTTCCGCCTTCCTTCCTAC 276**

**PX795202 ATATGGCATTCCCTCGAATAAATAATATGAGTTTTTGATTACTTCCGCCTTCCTTCCTAC 262**

**PX795203 ATATGGCATTCCCTCGAATAAATAATATGAGTTTTTGATTACTTCCGCCTTCCTTCCTAC 276**

**JF292393 ATATGGCATTCCCTCGAATAAATAATATGAGTTTTTGATTACTTCCGCCTTCCTTCCTAC 262**

**PX795197 ATATGGCATTTCCACGAATAAATAACATGAGTTTTTGATTACTCCCACCTTCCTTCCTAC 262**

**PX795196 ATATGGCATTTCCACGAATAAATAACATGAGTTTTTGATTACTCCCACCTTCCTTCCTAC 279**

**PX795194 ATATGGCATTTCCACGAATAAATAACATGAGTTTTTGATTACTCCCACCTTCCTTCCTAC 270**

**JF292415 ATATGGCATTTCCACGAATAAATAACATGAGTTTTTGATTACTCCCACCTTCCTTCCTAC 270**

**PX795195 ATATGGCATTTCCACGAATAAATAACATGAGTTTTTGATTACTCCCACCTTCCTTCCTAC 273**

**JF292338 ACATAGCATTCCCCCGAATAAATAATATGAGCTTCTGACTCCTTCCCCCCTCTTTTTTAC 278**

**JF292368 ACATGGCCTTCCCTCGAATAAACAATATAAGCTTCTGGCTCCTGCCCCCTTCCTTCTTAC 264**

**JF292353 ATATGGCCTTCCCTCGAATGAACAACATAAGCTTCTGGCTCCTGCCCCCCTCCTTCCTAC 258**

*** ** * * ** * * * * * * ****

**KJ590087 GTCAAATAATCTAATCAAGGCACAAAACTTAG-----TAAAACAT-AAGACT-TTACCTT 271**

**JF292297 TGCTACTCGCCTCATCAGGCGTTGAAGCAGGAGCTGGAACAGGGTGAACAGTGTACCCAC 324**

**JF292324 TGCTACTCGCCTCATCAGGTGTTGAAGCAGGGGCTGGAACAGGATGGACAGTATACCCGC 340**

**JF292311 TGCTACTCGCCTCATCAGGTGTTGAAGCAGGGGCTGGGACAGGGTGAACAGTATATCCAC 339**

**JF292339 TACTACTAGCTTCATCCGGTGTTGAAGCAGGTGCAGGAACTGGGTGAACTGTTTACCCAC 359**

**PX795198 TATTGCTTGCCTCCTCTGGAGTAGAAGCAGGGGCAGGAACAGGATGAACTGTATATCCAC 322**

**PX795199 TATTGCTTGCCTCCTCTGGAGTAGAAGCAGGGGCAGGAACAGGATGAACTGTATATCCAC 322**

**PX795204 TATTGCTTGCCTCCTCTGGAGTAGAAGCAGGGGCAGGAACAGGATGAACTGTATATCCAC 324**

**PX795205 TATTGCTTGCCTCCTCTGGAGTAGAAGCAGGGGCAGGAACAGGATGAACTGTATATCCAC 325**

**PX795200 TATTGCTTGCCTCCTCTGGAGTAGAAGCAGGGGCAGGAACAGGATGAACTGTATATCCAC 322**

**PX795201 TATTGCTTGCCTCCTCTGGAGTAGAAGCAGGGGCAGGAACAGGATGAACTGTATATCCAC 336**

**PX795202 TATTGCTTGCCTCCTCTGGAGTAGAAGCAGGGGCAGGAACAGGATGAACTGTATATCCAC 322**

**PX795203 TATTGCTTGCCTCCTCTGGAGTAGAAGCAGGGGCAGGAACAGGATGAACTGTATATCCAC 336**

**JF292393 TATTGCTTGCCTCCTCTGGAGTAGAAGCAGGGGCAGGAACAGGATGAACTGTATATCCAC 322**

**PX795197 TACTGCTCGCCTCATCTGGAGTTGAAGCGGGGGCAGGAACAGGATGAACTGTCTACCCAC 322**

**PX795196 TACTGCTCGCCTCATCTGGAGTTGAAGCGGGGGCAGGAACAGGATGAACTGTCTACCCAC 339**

**PX795194 TACTGCTCGCCTCATCTGGAGTTGAAGCGGGGGCAGGAACAGGATGAACTGTCTACCCAC 330**

**JF292415 TACTGCTCGCCTCATCTGGAGTTGAAGCGGGGGCAGGAACAGGATGAACTGTCTACCCAC 330**

**PX795195 TACTGCTCGCCTCATCTGGAGTTGAAGCGGGGGCAGGAACAGGATGAACTGTCTACCCAC 333**

**JF292338 TGCTACTAGCCTCCTCCGGAGTCGAAGCCGGGGCCGGAACAGGATGAACTGTTTATCCTC 338**

**JF292368 TCCTGCTTGCCTCCTCTGGGGTTGAAGCGGGAGCGGGGACAGGATGAACTGTTTACCCGC 324**

**JF292353 TTCTGCTCGCCTCCTCCGGAGTTGAAGCAGGAGCAGGAACAGGGTGAACTGTCTACCCGC 318**

*** * ** * ** * * * * * * ****

**KJ590087 CGGTTGGGGCGACCATGGAGGAAAAAACAGCCTCCAAGTGGATTGGACAC----ATCCCA 327**

**JF292297 CCCTTGCAGGAAACCTGGCACACGCGGGAGCCTCTG--TAGACTTAACAATCTTCTCCCT 382**

**JF292324 CACTTGCGGGTAACCTGGCGCATGCAGGAGCTTCTG--TAGATTTAACTATCTTCTCCCT 398**

**JF292311 CCCTTGCAGGAAACCTCGCACATGCAGGAGCTTCCG--TAGATTTAACTATTTTCTCCCT 397**

**JF292339 CACTCGCTGGAAATCTTGCACACGCAGGAGCATCTG--TAGATTTAACTATCTTCTCCCT 417**

**PX795198 CCCTTGCTGGAAACCTCGCACATGCCGGGGCTTCTG--TAGATTTAACTATTTTCTCCCT 380**

**PX795199 CCCTTGCTGGAAACCTCGCACATGCCGGGGCTTCTG--TAGATTTAACTATTTTCTCCCT 380**

**PX795204 CCCTTGCTGGAAACCTCGCACATGCCGGGGCTTCTG--TAGATTTAACTATTTTCTCCCT 382**

**PX795205 CCCTTGCTGGAAACCTCGCACATGCCGGGGCTTCTG--TAGATTTAACTATTTTCTCCCT 383**

**PX795200 CCCTTGCTGGAAACCTCGCACATGCCGGGGCTTCTG--TAGATTTAACTATTTTCTCCCT 380**

**PX795201 CCCTTGCTGGAAACCTCGCACATGCCGGGGCTTCTG--TAGATTTAACTATTTTCTCCCT 394**

**PX795202 CCCTTGCTGGAAACCTCGCACATGCCGGGGCTTCTG--TAGATTTAACTATTTTCTCCCT 380**

**PX795203 CCCTTGCTGGAAACCTCGCACATGCCGGGGCTTCTG--TAGATTTAACTATTTTCTCCCT 394**

**JF292393 CCCTTGCTGGAAACCTCGCACATGCCGGGGCTTCTG--TAGATTTAACTATTTTCTCCCT 380**

**PX795197 CTTTAGCCGGAAACCTAGCACATGCCGGAGCTTCTG--TAGATTTAACTATTTTCTCTCT 380**

**PX795196 CTTTAGCCGGAAACCTAGCACATGCCGGAGCTTCTG--TAGATTTAACTATTTTCTCTCT 397**

**PX795194 CTTTAGCCGGAAACCTAGCACATGCCGGAGCTTCTG--TAGATTTAACTATTTTCTCTCT 388**

**JF292415 CTTTAGCCGGAAACCTAGCACATGCCGGAGCTTCTG--TAGATTTAACTATTTTCTCTCT 388**

**PX795195 CTTTAGCCGGAAACCTAGCACATGCCGGAGCTTCTG--TAGATTTAACTATTTTCTCTCT 391**

**JF292338 CCCTTGCTGGAAACCTTGCACATGCCGGAGCTTCCG--TAGACCTAACCATTTTCTCATT 396**

**JF292368 CTCTTGCGGGCAACCTCGCACATGCAGGGGCCTCCG--TAGACTTAACCATCTTTTCCCT 382**

**JF292353 CTCTTGCCGGCAACCTCGCACATGCAGGGGCCTCCG--TAGATTTAACTATCTTTTCCCT 376**

*** * * * * * * * ** ** * ** ** ****

**KJ590087 AAAACCAAGAGAAACATCTCCACGTCA-------CAGAACATCTGACCAATTATGATCCG 380**

**JF292297 ACACCTAGCAGGTGTATCATCAATTCTTGCCTCAATCAACTTCATCACAACCATTATTAA 442**

**JF292324 ACACTTGGCAGGTGTATCATCAATTCTTGCTTCCATCAACTTCATTACAACTATCATCAA 458**

**JF292311 ACATCTAGCAGGTGTCTCATCAATTCTTGCATCTATTAATTTCATTACAACTATTATTAA 457**

**JF292339 TCATCTCGCAGGTGTCTCATCTATTCTAGGGGCCATTAATTTTATTACAACTATTATTAA 477**

**PX795198 TCATCTTGCAGGGGTATCATCCATTCTAGGAGCCATTAATTTTATTACAACCATTATTAA 440**

**PX795199 TCATCTTGCAGGGGTATCATCCATTCTAGGAGCCATTAATTTTATTACAACCATTATTAA 440**

**PX795204 TCATCTTGCAGGGGTATCATCCATTCTAGGAGCCATTAATTTTATTACAACCATTATTAA 442**

**PX795205 TCATCTTGCAGGGGTATCATCCATTCTAGGAGCCATTAATTTTATTACAACCATTATTAA 443**

**PX795200 TCATCTTGCAGGGGTATCATCCATTCTAGGAGCCATTAATTTTATTACAACCATTATTAA 440**

**PX795201 TCATCTTGCAGGGGTATCATCCATTCTAGGAGCCATTAATTTTATTACAACCATTATTAA 454**

**PX795202 TCATCTTGCAGGGGTATCATCCATTCTAGGAGCCATTAATTTTATTACAACCATTATTAA 440**

**PX795203 TCATCTTGCAGGGGTATCATCCATTCTAGGAGCCATTAATTTTATTACAACCATTATTAA 454**

**JF292393 TCATCTTGCAGGGGTATCATCCATTCTAGGAGCCATTAATTTTATTACAACCATTATTAA 440**

**PX795197 TCACTTGGCAGGGGTATCATCTATTCTAGGGGCCATTAACTTTATCACAACTATTATTAA 440**

**PX795196 TCACTTGGCAGGGGTATCATCTATTCTAGGGGCCATTAACTTTATCACAACTATTATTAA 457**

**PX795194 TCACTTGGCAGGGGTATCATCTATTCTAGGGGCCATTAACTTTATCACAACTATTATTAA 448**

**JF292415 TCACTTGGCAGGGGTATCATCTATTCTAGGGGCCATTAACTTTATCACAACTATTATTAA 448**

**PX795195 TCACTTGGCAGGGGTATCATCTATTCTAGGGGCCATTAACTTTATCACAACTATTATTAA 451**

**JF292338 ACATCTTGCAGGAGTCTCATCCATTCTAGGGGCCATCAACTTTATTACAACTATTATTAA 456**

**JF292368 ACACCTTGCAGGAGTTTCGTCTATTCTAGGGGCCATTAATTTTATTACAACAATTATTAA 442**

**JF292353 TCATCTTGCAGGAGTTTCATCTATTCTTGGGGCCATTAACTTTATTACAACAATTATTAA 436**

*** ** ** * ** ** * *** ** ****

**KJ590087 GTTAAAAAACCGATCAACGGACC------AAGTTACCCTAGGGATAACAGCGCAATCCTC 434**

**JF292297 TATAAAACCACCATCTATCTCCCAATACCAAACACCCTTATTTGTCTGATCTGTGATGAT 502**

**JF292324 CATAAAACCGCCTGCTATTTCCCAGTATCAAACACCTTTATTTGTTTGATCTGTAATAAT 518**

**JF292311 TATGAAACCCCCAGCTATTTCACAATATCAAACACCTTTATTTGTCTGATCAGTAATGAT 517**

**JF292339 CATAAAACCCCCAGCCATCTCTCAATACCAAACTCCTTTATTCGTATGAGCAGTTTTAAT 537**

**PX795198 CATAAAACCACCAGCAATTTCACAATATCAAACACCTTTATTTGTATGGGCTGTCTTAAT 500**

**PX795199 CATAAAACCACCAGCAATTTCACAATATCAAACACCTTTATTTGTATGGGCTGTCTTAAT 500**

**PX795204 CATAAAACCACCAGCAATTTCACAATATCAAACACCTTTATTTGTATGGGCCGTCTTAAT 502**

**PX795205 CATAAAACCACCAGCAATTTCACAATATCAAACACCTTTATTTGTATGGGCTGTCTTAAT 503**

**PX795200 CATAAAACCACCAGCAATTTCACAATATCAAACACCTTTATTTGTATGGGCTGTCTTAAT 500**

**PX795201 CATAAAACCACCAGCAATTTCACAATATCAAACACCTTTATTTGTATGGGCTGTCTTAAT 514**

**PX795202 CATAAAACCACCAGCAATTTCACAATATCAAACACCTTTATTTGTATGGGCTGTCTTAAT 500**

**PX795203 CATAAAACCACCAGCAATTTCACAATATCAAACACCTTTATTTGTATGGGCTGTCTTAAT 514**

**JF292393 CATAAAACCACCAGCAATTTCACAATATCAAACACCTTTATTTGTATGGGCTGTCTTAAT 500**

**PX795197 TATAAAACCCCCAGCAATTTCACAGTACCAAACACCTCTATTTGTATGAGCTGTACTAAT 500**

**PX795196 TATAAAACCCCCAGCAATTTCACAGTACCAAACACCTCTATTTGTATGAGCTGTACTAAT 517**

**PX795194 TATAAAACCCCCAGCAATTTCACAGTACCAAACACCTCTATTTGTATGAGCTGTACTAAT 508**

**JF292415 TATAAAACCCCCAGCAATTTCACAGTACCAAACACCTCTATTTGTATGAGCTGTACTAAT 508**

**PX795195 TATAAAACCCCCAGCAATTTCACAGTACCAAACACCTCTATTTGTATGAGCTGTACTAAT 511**

**JF292338 CATAAAACCCCCAGCCACATCACAATATCAAACACCCTTGTTTGTCTGAGCTGTCCTAAT 516**

**JF292368 CATAAAACCCCCCGCAATTTCACAATATCAGACTCCCCTATTTGTCTGAGCTGTCCTAAT 502**

**JF292353 TATGAAGCCCCCCGCAATCTCACAATATCAAACCCCCCTATTTGTCTGAGCCGTCCTAAT 496**

*** ** * * * * * * * * ***

**KJ590087 TCCAAGAGTCCATA---------------------------------------------- 448**

**JF292297 TACAGCGGTACTCCTACTCTTATCTCTTCCAGTACTAGCTGCGGGAATCACTATATTATT 562**

**JF292324 TACAGCAGTACTTCTACTTCTATCCCTACCAGTACTAGCTGCAGGAATCACTATATTATT 578**

**JF292311 TACAGCAGTACTCCTACTGCTTTCCCTCCCCGTACTAGCAGCAGGAATTACCATGCTATT 577**

**JF292339 TACAGCCGTACTCTTATTACTTTCCCTACCAGTACTAGCTGCTGGAATTACGATGCTACT 597**

**PX795198 TACAGCTGTTCTTCTATTATTATCTCTACCAGTACTGGCTGCCGGCATTACTATACTCCT 560**

**PX795199 TACAGCTGTTCTTCTATTATTATCTCTACCAGTACTGGCTGCCGGCATTACTATACTCCT 560**

**PX795204 TACAGCTGTTCTTCTATTATTATCTCTACCAGTACTGGCTGCCGGCATTACTATACTCCT 562**

**PX795205 TACAGCTGTTCTTCTATTATTATCTCTACCAGTACTGGCTGCCGGCATTACTATACTCCT 563**

**PX795200 TACAGCTGTTCTTCTATTATTATCTCTACCAGTACTGGCTGCCGGCATTACTATACTCCT 560**

**PX795201 TACAGCTGTTCTTCTATTATTATCTCTACCAGTACTGGCTGCCGGCATTACTATACTCCT 574**

**PX795202 TACAGCTGTTCTTCTATTATTATCTCTACCAGTACTGGCTGCCGGCATTACTATACTCCT 560**

**PX795203 TACAGCTGTTCTTCTATTATTATCTCTACCAGTACTGGCTGCCGGCATTACTATACTCCT 574**

**JF292393 TACAGCTGTTCTTCTATTATTATCTCTACCAGTACTGGCTGCCGGCATTACTATACTCCT 560**

**PX795197 TACAGCTGTACTTCTACTGCTATCTCTGCCCGTACTAGCCGCCGGCATTACAATACTTCT 560**

**PX795196 TACAGCTGTACTTCTACTGCTATCTCTGCCCGTACTAGCCGCCGGCATTACAATACTTCT 577**

**PX795194 TACAGCTGTACTTCTACTGCTATCTCTGCCCGTACTAGCCGCCGGCATTACAATACTTCT 568**

**JF292415 TACAGCTGTACTTCTACTGCTATCTCTGCCCGTACTAGCCGCCGGCATTACAATACTTCT 568**

**PX795195 TACAGCTGTACTTCTACTGCTATCTCTGCCCGTACTAGCCGCCGGCATTACAATACTTCT 571**

**JF292338 TACAGCCGTTCTCCTTCTACTAGCCCTACCAGTACTAGCCGCTGGTATTACAATACTCTT 576**

**JF292368 TACAGCCGTCCTACTACTACTATCCCTCCCAGTCCTAGCTGCTGGCATCACAATACTTCT 562**

**JF292353 TACAGCCGTCCTCCTACTACTATCCCTCCCAGTTTTAGCCGCTGGTATCACAATGCTTCT 556**

*** ** ** ***

**KJ590087 -TCGACGAGGGGGTTTACGACCTCGATGTTGGATCA----------GGACATCCTAATGG 497**

**JF292297 AACAGACCGAAATTTAAACACAACCTTCTTCGACCCTGCTGGAGGAGGAGACCCAATCCT 622**

**JF292324 AACAGACCGAAACTTAAACACAACTTTCTTTGATCCTGCTGGTGGGGGAGACCCAATTCT 638**

**JF292311 AACGGACCGAAATCTAAATACTACATTCTTTGACCCTGCCGGGGGAGGGGACCCAATCCT 637**

**JF292339 AACAGATCGGAACCTCAATACTACATTCTTTGATCCGGCAGGAGGGGGTGACCC------ 651**

**PX795198 AACAGATCGAAACCTAAATACTACATTCTTTGATCCTGCAGGCGGAGGAGATCCTATTTT 620**

**PX795199 AACAGATCGAAACCTAAATACTACATTCTTTGATCCTGCAGGCGGAGGAGATCCTATTTT 620**

**PX795204 AACAGATCGAAACCTAAATACTACATTCTTTGATCCTGCAGGCGGAGGAGATCCTATTTT 622**

**PX795205 AACAGATCGAAACCTAAATACTACATTCTTTGATCCTGCAGGCGGAGGAGATCCTATTTT 623**

**PX795200 AACAGATCGAAACCTAAATACTACATTCTTTGATCCTGCAGGCGGAGGAGATCCTATTTT 620**

**PX795201 AACAGATCGAAACCTAAATACTACATTCTTTGATCCTGCAGGCGGAGGAGATCCTATTTT 634**

**PX795202 AACAGATCGAAACCTAAATACTACATTCTTTGATCCTGCAGGCGGAGGAGATCCTATTTT 620**

**PX795203 AACAGATCGAAACCTAAATACTACATTCTTTGATCCTGCAGGCGGAGGAGATCCTATTTT 634**

**JF292393 AACAGATCGAAACCTAAATACTACATTCTTTGATCCTGCAGGCGGAGGAGATCCTATTTT 620**

**PX795197 AACAGACCGAAATCTAAATACTACATTCTTCGACCCCGCAGGGGGAGGAGACCCCATCCT 620**

**PX795196 AACAGACCGAAATCTAAATACTACATTCTTCGACCCCGCAGGGGGAGGAGACCCCATCCT 637**

**PX795194 AACAGACCGAAATCTAAATACTACATTCTTCGACCCCGCAGGGGGAGGAGACCCCATCCT 628**

**JF292415 AACAGACCGAAATCTAAATACTACATTCTTCGACCCCGCAGGGGGAGGAGACCCCATCCT 628**

**PX795195 AACAGACCGAAATCTAAATACTACATTCTTCGACCCCGCAGGGGGAGGAGACCCCATCCT 631**

**JF292338 AACAGACCGAAACCTAAACACCACATTCTTTGACCCTTCAGGTGGAGGAGACCCAATTCT 636**

**JF292368 AACAGACCGAAACTTAAACACCACCTTCTTTGACCCCGCAGGGGGAGGGGACCCCATTCT 622**

**JF292353 AACAGACCGAAACTTAAATACTACCTTCTTTGACCCTGCAGGGGGAGGCGACCCCATTCT 616**

*** * * * ** * * ** ** * ** * ****

**KJ590087 TGCAGCCGCTATTAAGGGTTCGTTTGTTCAACGATT 533**

**JF292297 TTATCAACACCTCTTTGATTCTTCGGTCA------- 651**

**JF292324 TTACCAACACCTC----------------------- 651**

**JF292311 CTACCAGCATCTCT---------------------- 651**

**JF292339 ------------------------------------ 651**

**PX795198 ATATCAACATCTTTTCTGATTCTTCCGTCAC----- 651**

**PX795199 ATATCAACATCTTTTCTGATTCTTCCGTCAC----- 651**

**PX795204 ATATCAACATCTTTTCTGATTCTTCGGTCACCC--- 655**

**PX795205 ATATCAACATCTTTTCTGATTCTTCGGT-------- 651**

**PX795200 ATATCAACATCTTTTCTGATTCTTCGGTCAC----- 651**

**PX795201 ATATCAACATCTTTTCTGATTCTTCGGTCACCCT-- 668**

**PX795202 ATATCAACATCTTTTCTGATTCTTCGGTCAC----- 651**

**PX795203 ATATCAACATCTTTTCTGATTCTTCGGTCACCCT-- 668**

**JF292393 ATATCAACATCTTTTCTGATTCTTCGGTCAC----- 651**

**PX795197 ATATCAACAGCTTTTCTGATTCTTCGGTCAC----- 651**

**PX795196 ATATCAACACCTTT---------------------- 651**

**PX795194 ATATCAACACCTTTTCTGATTCT------------- 651**

**JF292415 ATATCAACACCTTTTCTGATTCT------------- 651**

**PX795195 ATATCAACACCTTTTCTGAT---------------- 651**

**JF292338 TTACCAGCATCTTTTCTGATTCTTTGGGCACC---- 668**

**JF292368 TTACCAACACCTTTTCTGATTCTTCGGTC------- 651**

**JF292353 TTACCAACACCTTTTCTGATTCTTCGGTCACCCTG- 651**

**Figure S3.** Multiple sequence alignment of imported fish fillets in Egyptian markets, catfish species sequences from GenBank, and *Devario aequipinnatus sequence*, generated by Clustal Omega to construct the phylogenetic tree. Identities and similarities are indicated by star (*) and gaps are indicated by dashes (-).
