# Supplementary material for: A case study for application of DNA barcoding in identifying species of some imported frozen fish fillets in Egypt
Source: Sci Rep. 2026 Jun 26;16:19612. doi: 10.1038/s41598-026-58341-0 (PMC13309559; doi:10.1038/s41598-026-58341-0)
Supplement: Supplementary file 2 — Supplementary Material 2 [file 41598_2026_58341_MOESM2_ESM.docx]

**A case study for application of DNA barcoding in identifying species of some imported frozen fish fillets in Egypt**

Nermeen Y. Abass^a*^

^a^ Department of Agricultural Botany, Faculty of Agriculture Saba-Basha, Alexandria University, Alexandria City, P.O. Box 21531, Egypt

*Corresponding author: + 2 03 5831646; fax: + 2 035832008

E-mail address: [n.y.abass@alexu.edu.eg](mailto:n.y.abass@alexu.edu.eg)

### Table S1. DNA sequence analysis, base composition, and the average AT and GC nucleotide composition in the studied frozen fish fillets samples.

| **Samples** | **A** | **T** | **G** | **C** | **Total**  **(bp)** | **AT (%)** | **GC (%)** |
| --- | --- | --- | --- | --- | --- | --- | --- |
| Fish fillet_1 | 167 | 191 | 121 | 172 | 651 | 55.15 | 44.85 |
| Fish fillet_2 | 169 | 190 | 120 | 172 | 651 | 55.30 | 44.70 |
| Fish fillet_3 | 169 | 191 | 119 | 172 | 651 | 54.84 | 45.16 |
| Fish fillet_4 | 169 | 188 | 120 | 174 | 651 | 57.76 | 42.24 |
| Fish fillet_5 | 168 | 208 | 113 | 162 | 651 | 57.76 | 42.24 |
| Fish fillet_6 | 168 | 208 | 113 | 162 | 651 | 57.76 | 42.24 |
| Fish fillet_7 | 168 | 208 | 114 | 161 | 651 | 57.78 | 42.22 |
| Fish fillet_8 | 171 | 215 | 117 | 165 | 668 | 57.76 | 42.24 |
| Fish fillet_9 | 168 | 208 | 114 | 161 | 651 | 57.78 | 42.22 |
| Fish fillet_10 | 171 | 215 | 117 | 165 | 668 | 57.40 | 42.60 |
| Fish fillet_11 | 168 | 208 | 115 | 164 | 655 | 57.76 | 42.24 |
| Fish fillet_12 | 167 | 209 | 116 | 159 | 651 | 55.15 | 44.85 |
| **Average** |  |  |  |  |  | **56.84** | **43.16** |
